# Modelling of oscillatory cortisol response in horses using a Bayesian population approach for evaluation of dexamethasone suppression test protocols

**DOI:** 10.1007/s10928-018-09617-0

**Published:** 2019-01-23

**Authors:** Felix Held, Carl Ekstrand, Marija Cvijovic, Johan Gabrielsson, Mats Jirstrand

**Affiliations:** 1 Fraunhofer-Chalmers Centre, Chalmers Science Park, Gothenburg, Sweden; 2 Department of Mathematical Sciences, Chalmers University of Technology and University of Gothenburg, Gothenburg, Sweden; 3 Department of Biomedical Sciences and Veterinary Public Health, Swedish University of Agricultural Sciences, Uppsala, Sweden

**Keywords:** Cortisol, Dexamethasone suppression test, Bayesian inference, Oscillating baseline, Turnover model, Inter-individual variability, NLME

## Abstract

Cortisol is a steroid hormone relevant to immune function in horses and other species and shows a circadian rhythm. The glucocorticoid dexamethasone suppresses cortisol in horses. Pituitary pars intermedia dysfunction (PPID) is a disease in which the cortisol suppression mechanism through dexamethasone is challenged. Overnight dexamethasone suppression test (DST) protocols are used to test the functioning of this mechanism and to establish a diagnosis for PPID. However, existing DST protocols have been recognized to perform poorly in previous experimental studies, often indicating presence of PPID in healthy horses. This study uses a pharmacokinetic/pharmacodynamic (PK/PD) modelling approach to analyse the oscillatory cortisol response and its interaction with dexamethasone. Two existing DST protocols were then scrutinized using model simulations with particular focus on their ability to avoid false positive outcomes. Using a Bayesian population approach allowed for quantification of uncertainty and enabled predictions for a broader population of horses than the underlying sample. Dose selection and sampling time point were both determined to have large influence on the number of false positives. Advice on pitfalls in test protocols and directions for possible improvement of DST protocols were given. The presented methodology is also easily extended to other clinical test protocols.

**Electronic supplementary material:**

The online version of this article (10.1007/s10928-018-09617-0) contains supplementary material, which is available to authorized users.

## Introduction

Dexamethasone and other glucocorticoids are commonly used in equine medicine for the treatment of diseases and clinical testing, *e.g.*, the dexamethasone suppression test (DST) [1–3]. In healthy horses, dexamethasone suppresses the cortisol response [4, 5]. The mechanism is challenged in horses affected by pituitary pars intermedia dysfunction (PPID) [6]. PPID is an age-related degenerative disease leading to a loss of dopaminergic neurons affecting the pars intermedia of the pituitary gland [7]. Similarities of PPID to Parkinson’s disease in humans have been found [8]. The most prevalent clinical signs of PPID in horses are hair coat abnormalities, laminitis and muscle atrophy [9]. The prevalence of PPID in horses aged more than 15 years is 21% [10].

The idea behind the DST is to observe whether the cortisol response is suppressed below a threshold after dexamethasone administration [1]. Different test protocols have been published under the name *overnight DST* [1, 11]. They differ in dexamethasone administration time as well as waiting-time until cortisol measurement. Both are designed as single-point observation test protocols which have limitations, since DST results may indicate presence of PPID in healthy individuals due to a circadian rhythm in cortisol production in addition to inter-individual variability [12]. In horses, plasma cortisol concentration displays an apparently symmetric circadian variation with peak concentration in the morning and nadir concentration in the afternoon/evening [13, 14].

Pharmacokinetic (PK) and pharmacodynamic (PD) modelling is done to quantify the relationship between a physiological response and drug exposure [15]. The disposition of dexamethasone in horses has been characterized in several studies [12, 16–20]. The relationship between dexamethasone and cortisol response has also been characterised by turnover modelling with inhibition of a constant [21] as well as an oscillating turnover rate [12]. Those studies focused on modelling individual concentration–time as well as response-time courses. Modelling studies have not estimated potential variability between animals.

A non-linear mixed-effects (NLME) approach allows simultaneous regression of all individuals and time courses [22]. A merit of this technique is the estimation of inter-individual variability (IIV) directly from data [23]. The use of mixed effects models has historically not been extensively used in veterinary science but recently has been given more attention [24]. Mixed-effects models can be formulated as hierarchical Bayesian models [25]. The Bayesian approach allows for incorporation of prior knowledge as well as modelling of all sources of variability and uncertainty. This allows for explicit propagation of uncertainty in parameter estimates and residual variability to predictions made using the final adjusted model [22]. Including all sources of uncertainty and simulating predictions from the full model, as is straight-forward in the Bayesian approach, increases the credibility of the predictions. Prior information about parameter estimates and variability is available from an earlier study [12].

In this study, we sought to analyse data from a previous study [12] by means of a NLME approach to investigate the IIV. We then used the adjusted model to scrutinize DST protocol designs, define weaknesses in two proposed test protocols and give directions for test improvement.

## Materials and methods

### Experimental setup and analytical method

Six Standardbred horses (four mares and two geldings) 6–20 years old and weighing 430–584 kg were included in the study and assigned to a randomised crossover design including four treatments and four periods. Each treatment started with an intravenous bolus dose immediately followed by 3 h of constant rate infusion of dexamethasone 21-phosphate disodium salt (Dexadreson 2 mg mL^−1^, Intervet AB, Sollentuna, Sweden). The dose levels were (bolus + infusion) 0.1 + 0.07 μg kg^−1^, 1 + 0.7 μg kg^−1^ and 10 + 7 μg kg^−1^ dexamethasone. For the control level 0.9% saline was used. Before the bolus dose (time = 0) a pre-dose blood sample was drawn. Additional blood samples were drawn during and after infusion at hours 1, 2, 3, 4, 5, 6, 9, 12, 18, 24, 36 and 48. A minimum of a 1 week wash-out period was allowed between drug treatments. The study was approved by the Ethics Committee for Animal Experiments, Uppsala, Sweden (C333/11). Total plasma dexamethasone and cortisol concentrations were analysed and quantified using Ultra High Performance Liquid Chromatography-Tandem Mass Spectrometry (UHPLC-MS/MS). The analytical method was described before elsewhere [21].

### Dexamethasone exposure

**Fig. 1:**
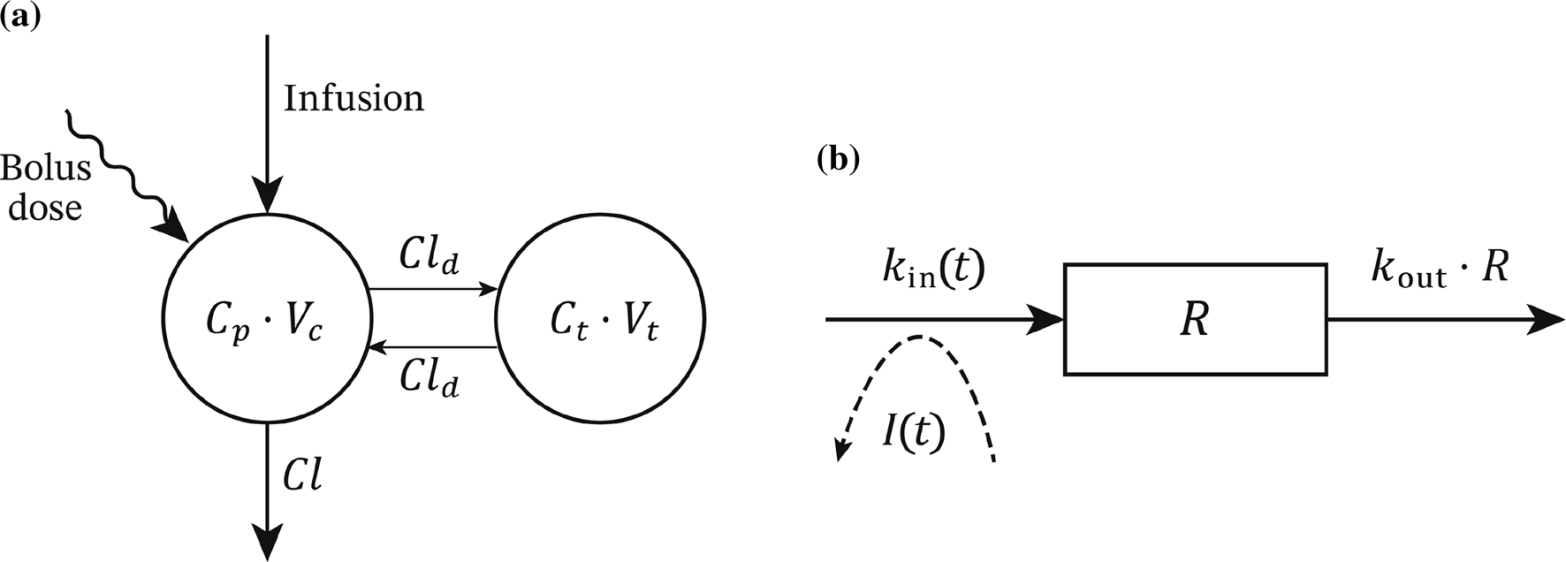
**a** Dexamethasone disposition model after the bolus + constant rate infusion regimens. **b** Conceptual model of the cortisol turnover with an oscillating baseline k_in_(t)

A two-compartment model (Eq. 1, Fig. 1a) was fitted to experimental dexamethasone-time course data.

1 $$\left\{{\begin{array}{ll}V_{c} \frac{{\text{d}}C_{p}}{\text{d}t} = \text{Inf}(t) - Cl \cdot C_{p} + Cl_{d} \left( C_{t} - C_{p} \right), & C_{p} (0) = \frac{\text{D}}{V_{c}} \\{V_{t} \frac{{{\text{d}}C_{t} }}{{{\text{d}}t}} = Cl_{d} \left( {C_{p} - C_{t} } \right),} & C_{t} (0) = 0\end{array}}\right.$$

*C*_*p*_ and *C*_*t*_ denote drug concentration in central (plasma) and peripheral compartments. *V*_*c*_, *V*_*t*_, *Cl* and *Cl*_*d*_ denote, respectively, the central and peripheral volumes, plasma clearance and inter-compartmental distribution parameter. Inf(*t*) represents the constant rate infusion regimen and *D* is the bolus dose administered at time *t*  =  0.

### Cortisol turnover

Cortisol was modelled by a turnover model (Eqs. 2–4, Fig. 1b).

2 $$\frac{{{\text{d}}R}}{{{\text{d}}t}} = k_{\text{in}} (t) \cdot I(t) - k_{\text{out}} R,\quad R(0) = R_{\text{eq}} (t = 0,C_{{p,\,{\text{eq}}}} = 0)$$

*R* is cortisol concentration, *k*_out_ the fractional turnover rate and *R*_eq_ stands for the expression in Eq. 5. The oscillatory behaviour of turnover rate was modelled by Eq. 3. Note that in the following *ω* = 2*π*/24 h^−1^.

3 $$k_{\text{in}}(t) = k_{\text{avg}} \cdot 
\left(1 + \alpha \cdot \cos\left(\omega\, (t - t_0)\right) \right)$$

*k*_avg_ is positive and corresponds to the average turnover rate. *t*_0_ is the phase shift between − 12 and 12 h, and *α*, a number between 0 and 1, describes the amplitude of the oscillations as a proportion of *k*_avg_. Choosing *α* this way ensures positivity of the turnover rate for all choices of parameters. The period was fixed at 24 h. The inhibitory dexamethasone mechanism function was modelled as

4 $$I(t) = 1 - \frac{{I_{ \text{max} }\,C_{p}^{n} (t)}}{{IC_{50}^{n} + C_{p}^{n} (t)}}\,,$$

where *I*_max_ is maximum inhibitory capacity, *IC*_50_ the potency of dexamethasone and *n* is a Hill exponent. This model is a modified version of the single cosine model presented in [26].

### Cortisol concentration under constant dexamethasone exposure

An oscillating turnover rate leads to oscillating cortisol concentration. Keeping dexamethasone exposure in Eq. 4 constant at a fixed concentration *C*_*p*__, eq_, the cortisol response is given by

5 $$R_{\text{eq}} (t,C_{{p,\,{\text{eq}}}} ) = A + B \cdot { \cos }\left( {\omega\, (t - C)} \right)\,,$$

where

6 $$\begin{aligned} 
A & = 
\frac{k_{\text{avg}}}{k_{\text{out}}}
\cdot \left(1 - 
\frac{I_{\text{max}}\,C_{p,\,\text{eq}}^n}{IC_{50}^n + C_{p,\,\text{eq}}^n }
\right)\,, \\ 
B & = 
\frac{k_{\text{avg}} \cdot \alpha}{\sqrt{k_{\text{out}}^2 + \omega^2}} 
\cdot \left(1 - 
\frac{I_{\text{max}}\,C_{p,\,\text{eq}}^n}{IC_{50}^n + C_{p,\,\text{eq}}^n } 
\right)\,, \\ 
C & = \frac{1}{\omega}\,\arctan
\left(
\frac{k_{\text{out}}\,\sin(\omega t_0) + \omega\,\cos(\omega t_0)}{k_{\text{out}}\,\cos(\omega t_0) - \omega\,\sin(\omega t_0)} \right)\,.
\end{aligned}$$

*A* describes the average cortisol response, *B* the amplitude and *C* the phase shift of the oscillation. The model predicts only changes in the average cortisol response and amplitude due to changes in dexamethasone exposure. A derivation of *R*_eq_ in Eq. 5 as well as for *A*, *B* and *C* in Eq. 6 can be found in the Appendix. The ideas are similar to the calculations presented in Krzyzanski et al. [27].

### Residual error variance model

Kinetic data was modelled on a log scale. For the drug exposure model in Eq. 1 it was assumed that

7 $${ \log }\left( {C_{p} \left( {t_{ij} } \right)} \right) = { \log }\left( {\widehat{{C_{p} }}\left( {t_{ij} } \right)} \right) + e_{ij} .$$

For the cortisol response model in Eq. 2 a combined error model with proportional and additive error was assumed. This was described by

8 $$R\left( {t_{ij} } \right) = \hat{R}\left( {t_{ij} } \right)\left( {1 + s_{ij}^{(1)} } \right) + s_{ij}^{(2)} .$$

Here, *C*_*p*_(*t*_*ij*_) and *R*(*t*_*ij*_) are the *j*th measurement of the plasma concentration of dexamethasone in the central compartment and cortisol, respectively, measured for subject *i* at time point *t*_*ij*_. $\widehat{{C_{p} }}(t_{ij} )$ and $\hat{R}(t_{ij} )$ are the predicted concentrations for subject *i* at time point *t*_*ij*_. *e*_*ij*_ as well as $s_{ij}^{(1)}$ and $s_{ij}^{(2)}$ were assumed to be normally distributed with zero mean and respective standard deviations *ε* as well as $\sigma_{1}$ and $\sigma_{2}$.

### Statistical parameter model

IIV was modelled by making the following assumptions about the distribution of the parameters in Eqs. 1–4. The process of deciding which parameters were modelled with correlation is described in the Supplementary.

All parameters involved in the description of dexamethasone exposure were modelled independently log-normally distributed, i.e.,

9 $$\begin{aligned}
\log(\theta) & \sim {\text{Log-Normal}}\,(\mu ,\tau^{2})\,, \\
\mu & \sim {\text{Normal}}\,(m_{0} ,1)\,, \\ 
\tau & \sim {\text{Student-t}}\,(4,s_{0} ,0.25)\,,
\end{aligned}$$

where *m*_0_ and *s*_0_ are prior parameters and *θ* stands for *Cl*, *Cl*_*d*_, *V*_*c*_ and *V*_*t*_. Some parameters in the cortisol turnover model were modelled with correlations as

10 $$\left( {\begin{array}{*{20}c} {{ \log }\left( {k_{\text{avg}} } \right)} \\ {{ \log }\left( {k_{\text{out}} } \right)} \\ {{ \log }\left( {IC_{50} } \right)} \\ {{\text{logit}}\left( \alpha \right)} \\ {{\text{logit}}\left( {\frac{{t_{0} + 12{\text{h}}}}{{24{\text{h}}}}} \right)} \\ \end{array} } \right) \sim {\text{Normal}}\left( {{\varvec{\upmu}},\,{\varvec{\Omega}}} \right),$$

where **Ω** = **LDL**^*T*^, $\varvec{D} = \text{diag}(\tau_{1}^{2} ,\;\tau_{2}^{2} ,\;\tau_{3}^{2} ,\;\tau_{4}^{2} ,\;\tau_{5}^{2} )$ and **L** is a lower-triangular matrix. In this representation the matrix **D** contains the variances and **LL**^*T*^ is the correlation matrix. In addition

11 $$\begin{aligned} 
n &\sim {\text{Normal}}\,(\mu_n ,\tau_n)\,, \\ 
I_{\text{max}} &\sim {\text{Logit-Normal}}\,(\mu_{{I_{ \text{max} } }} ,\tau_{{I_{ \text{max} } }} )\,.
\end{aligned}$$

Hyperparameters *μ* in Eqs. 10, 11 and the diagonal elements of **D** as well as *τ* in Eq. 11 are distributed as

12 $$\begin{aligned}
\mu &\sim {\text{Normal}}\,(m_0, v)\,, \\
\tau &\sim {\text{Student-t}}\,(4, s_0, 0.25)\;,\;\;\tau \geq 0\,.
\end{aligned}$$

where $m_{0}$ and $s_{0}$ are prior parameters, *v* = 2.5 for hyperparameters related to *α* as well as *t*_0_, and *v* = 1 otherwise. The three-parameter Student-t distributions used in Eqs. 9, 12 for non-negative *τ* are truncated distributions.

The correlation matrix **LL**^*T*^ was assumed to be distributed following a LKJ distribution [28] with concentration parameter 2. This is a prior for correlation matrices where samples resemble the identity matrix more closely for concentration parameters closer to 1. Residual-error-model standard deviations *ε* and $\sigma_{1}$ and $\sigma_{2}$ were assumed to be positive and were given half-Cauchy prior distributions [29] with scale 2.5. Prior parameters were estimated from a meta-analysis of Ekstrand et al. [12] as described in the Appendix.

### Analysis of the dexamethasone suppression test protocol

We simulated two different *overnight DST* protocols. Each consisted of a dexamethasone administration time and a sampling time on the following day. Cortisol concentration is analysed in the sampled blood plasma and the result of the DST is positive if concentration is above a prescribed threshold. The protocols analysed more closely are described in Dybdal et al. [1] (protocol A) and Frank et al. [11] (protocol B). Both protocols assume administration of 40 μg kg^−1^ of dexamethasone. The protocols differ in administration route. Protocol A assumes *intramuscular* (im) administration whereas protocol B assumes *intravenous* (iv) administration. Test starting times were at 9.00 a.m. (protocol B) and 5 p.m. (protocol A). Plasma sampling times for determination of cortisol concentration were after 19 h (protocol A) and 24 h (protocol B), respectively. In both protocols, the test is positive (indicating sick individuals) if measured cortisol concentration is above a threshold of 10 μg L^−1^.

The DST protocols were analysed in light of two different aspects. First, we performed a Monte Carlo study to visualise cortisol time courses for horses subjected to each protocol. A sample of 10,000 horses was simulated from the adjusted model. For this, residual variance parameters and hyper-parameters (N = 1000) were taken from the estimated posterior parameter distribution. Then, individual parameters (N = 10) were simulated from hyper-parameters and the distributions in Eqs. 9–11. Dexamethasone- and cortisol time courses were simulated under the two test protocols for the new subjects using Eqs. 1–4 as well as the measurement equation (Eq. 8). The investigated protocols assume administration of 40 μg kg^−1^ dexamethasone and the aim of this simulation was to determine whether this amount is necessary or if lower doses could be sufficient. Predicted cortisol concentration at sampling time was then used for further analysis.

These concentrations were then used in a second step to investigate both the *sensitivity* of the test, i.e., the probability that the test is positive for a sick subject, as well as the *specificity* of the test, i.e., the probability that the test is negative for a healthy subject [30]. The distributions of sensitivity and specificity were simulated through a combination of Monte Carlo and analytical steps. See the Appendix for the formulas used. In horses with PPID the mechanism for dexamethasone suppression of cortisol is challenged [6]. To quantify sensitivity, simulations from sick horses were needed. We hypothesized that dexamethasone has no suppression effect on sick individuals and therefore these horses were sampled at baseline. The studies reporting protocol A and B [1, 11] determined sensitivity and specificity experimentally and this analysis aimed to investigate if model predicted and experimentally determined values are aligned.

### Numerical analysis and parameter estimation

The software Stan version 2.18.0 [31] was used for parameter inference through the interface CmdStan. Stan implements the NUTS sampler [32] that uses Hamiltonian Monte Carlo (HMC) [33] for estimation of the posterior parameter distribution and allows models with differential equations. PK and PD parameters were estimated in two stages. First, PK parameters in Eq. 1 were estimated. Each individual’s PK parameters were then summarised and fixed to the respective conditional mean. In a second stage, the PD parameters in Eqs. 2–4 were estimated. In each stage, four Markov chains were started at random initial parameters around the prior parameter means. Each chain was run for 250 iterations in warm-up and sampling, respectively. This led to a total of N = 1000 samples from the posterior.

The convergence of the HMC algorithm was checked in multiple ways. Numerical divergences during parameter estimation were observed and appropriate choices about parameter distributions were made and Stan settings were tuned to reduce and avoid divergences [34]. The Gelman–Rubin $\hat{R}$ statistic [35] and trace plots were used to ensure proper mixing of the Markov chains. The effective sample size [25] was observed to be at least 10% of total samples size (N = 1000). The energy Bayesian fraction of missing information (E-BFMI) [36] was checked to ensure that the parameter space was properly and efficiently explored. No external validation data was available and therefore internal model checking was performed through posterior predictive checks (PPCs) [25]. These visualisations are similar to visual predictive checks (VPCs) [37]. However, PPCs include parameter uncertainty by simulating the response from the full estimated posterior distribution, whereas VPCs omit this. Estimated parameters were summarised by median and 95% credible intervals (CIs) [25]. Population predicted ranges were calculated as described in the Supplementary.

## Results

### Regression of experimental time courses

**Fig. 2:**
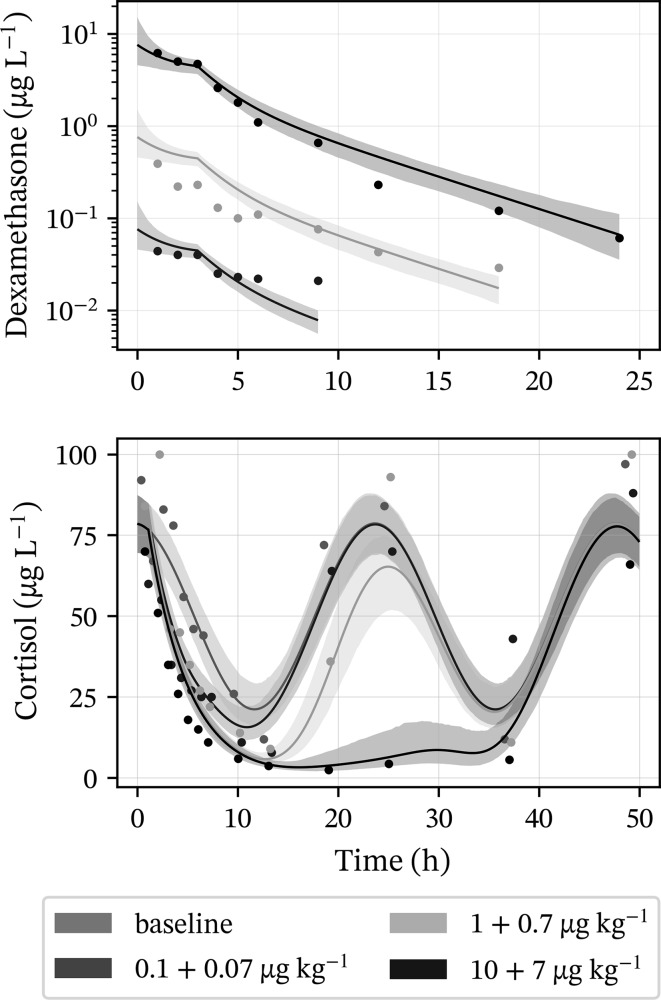
Dexamethasone and cortisol time course data and model predictions for a representative horse. Different dosing regimens are indicated by colour and respective dosing amounts are shown in the legend. Solid lines are time courses corresponding to the subject’s average parameters. Uncertainty in predicted time-courses is shown by shaded areas representing 95% of uncertainty

The drug exposure model captured dexamethasone exposure across three orders of magnitude (Figs. 2, S1). In general, within and between subject variability in the dexamethasone time courses were low, which suggests that exposure of dexamethasone does not confound the cortisol response. Observed and regressed dexamethasone-time courses following the three dosing regimens for a representative horse are shown in Fig. 2. The final population model parameters as well as their predicted population range are shown in Table 1. Summaries of individual parameters per horse are reported in the Supplementary (Table S1).

**Table 1:** Estimated population parameters, the IIV standard deviation and predicted population range for the dexamethasone model

Parameter (unit)	Typical value^a^		IIV std. dev.^a^		Predicted population range^a^	
*Cl* (L h^−1^ kg^−1^)	0.50	(0.43, 0.56)	0.071	(0.0036, 0.22)	0.50	(0.39, 0.63)
*Cl*_*d*_ (L h^−1^ kg^−1^)	0.16	(0.078, 0.33)	0.40	(0.032, 0.86)	0.16	(0.049, 0.55)
*V*_*c*_ (L kg^−1^)	1.2	(0.74, 1.7)	0.15	(0.013, 0.48)	1.2	(0.62, 2.1)
*V*_*t*_ (L kg^−1^)	0.76	(0.52, 1.0)	0.14	(0.013, 0.48)	0.76	(0.42, 1.3)

^a^Values reported as median and 95% credible interval

The observed cortisol response was well captured by the model (Figs. 2, S2). The largest variability in cortisol response was observed in baseline data. Increasing exposure to dexamethasone suppressed both the average cortisol response and the amplitude of oscillations (Fig. 2), which was furthermore captured by the model. The suppression of cortisol by dexamethasone was almost complete, which is also evident from the estimated values of *I*_max_ close to 1 (Table 2). The typical potency value was predicted to be 37 ng L^−1^ varying between 22 and 56 ng L^−1^, and the half-life of the cortisol response (ln (2)/*k*_out_) was predicted to be 2.0 h varying between 1.1 and 3.4 h. Observed and regressed cortisol response-time courses following the three dosing regimens and baseline for a representative horse are shown in Fig. 2. The final population model parameters as well as their predicted population range are shown in Table 2. Summaries of individual parameters per horse are reported in the Supplementary (Table S2).

**Table 2:** Estimated population parameters, the IIV standard deviation and predicted population range for the cortisol model

Parameter (unit)	Typical value^a^		IIV std. dev.^a^		Predicted population range^a^	
*k*_avg_ (μg L^−1^ h^−1^)	15	(11, 22)	0.36	(0.15, 0.67)	15	(6.4, 37)
*α* (unitless)	0.30	(0.13, 0.58)	1.1	(0.77, 1.7)	0.31	(0.035, 0.85)
*t*_0_ (h)	− 2.9	(− 4.3, − 1.3)	0.12	(0.0052, 0.37)	− 3.0	(− 5.1, − 0.095)
*k*_out_ (h^−1^)	0.34	(0.27, 0.46)	0.20	(0.080, 0.45)	0.34	(0.20, 0.62)
*I*_max_ (unitless)	0.99	(0.97, 1.0)	0.41	(0.050, 0.96)	0.94	(0.81, 0.98)
*IC*_50_ (ng L^−1^)	37	(22, 56)	0.56	(0.20, 0.97)	37	(9.1, 130)
*n* (unitless)	2.3	(1.5, 3.4)	0.39	(0.062, 0.99)	2.4	(1.1, 3.8)

^a^Values reported as median and 95% credible interval

Uncertainty in parameter estimates and IIV are included in the Bayesian posterior distribution and were readily analysed. Variability in typical values and IIV standard deviation was directly estimable. Predicted population ranges contain variability stemming from both uncertainty in population parameters and variability from the distributional assumptions in the section “Statistical parameter model”. Uncertainty in population parameters was moderate with larger amounts of variability observed in *Cl*_*d*_ (Table 1) as well as *α*, *t*_0_ and *IC*_50_ (Table 2). Uncertainty in IIV standard deviations was comparably larger, with 95% credible intervals often spanning double the magnitude of the median. For parameters in the dexamethasone model IIV was estimated to be small, with most credible intervals reaching close to zero. For *Cl* the smallest amount of variability (less than 0.2) was estimated whereas estimates for *Cl*_*d*_ were largest (less than 0.9). Predicted IIV for the parameters in the cortisol model was larger, particularly for parameters *k*_avg_, *α* and *IC*_50_, with credible intervals being clearly bounded from below. Estimated IIV for other parameters was considered to be small with most variability in the predicted population range stemming from uncertainty in estimated typical values.

After initial test runs and analysis of possible correlations between individual parameters, correlation estimates were included for *k*_avg_, *α*, *t*_0_, *k*_out_ and *IC*_50_. The resulting estimates can be found in the Supplementary (Table S3). Most correlation parameters showed only a slight if any tendency towards the positive or negative. Results suggest possible negative correlations between *k*_avg_ and *α*, *k*_out_ and *α*, and *IC*_50_ and *α*, but high uncertainty makes the estimates inconclusive.

### Simulation of cortisol response versus dexamethasone plasma concentration

**Fig. 3:**
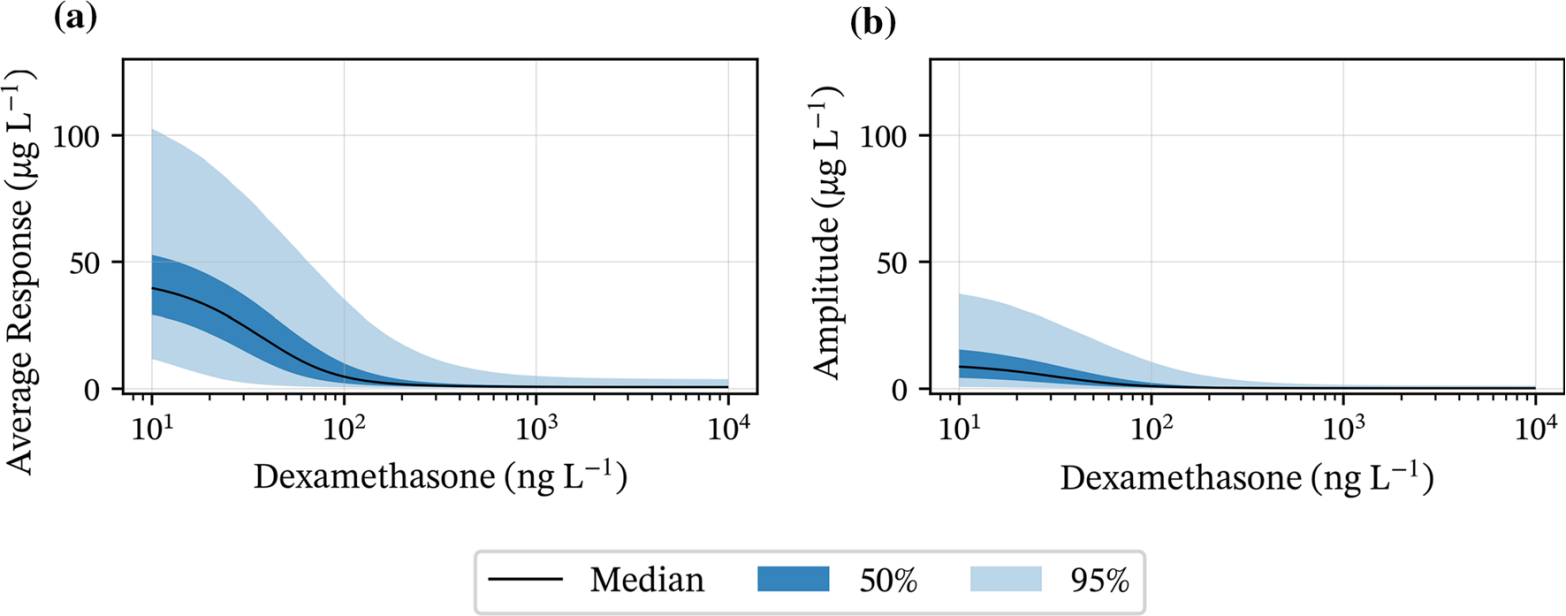
**a** Plot of the average response (A in Eq. 6). **b** Plot of the oscillation amplitude (B in Eq. 6). Variability is shown as shaded areas

Model simulations of the equilibrium dexamethasone-cortisol response relationship (Fig. 3a), and the amplitude of cortisol response (Fig. 3b) with increasing dexamethasone concentrations show that at concentrations around the potency value (about 20 to 50 ng L^−1^) small changes in dexamethasone plasma concentration produce disproportionally large changes in cortisol response and amplitude. At concentrations well above the potency value (> 100 ng L^−1^) small changes in dexamethasone plasma concentration produce small changes in cortisol response and amplitude. Note the almost complete suppression of cortisol response and its variability, with increasing dexamethasone concentrations.

### Simulation of two overnight DST protocols

**Fig. 4:**
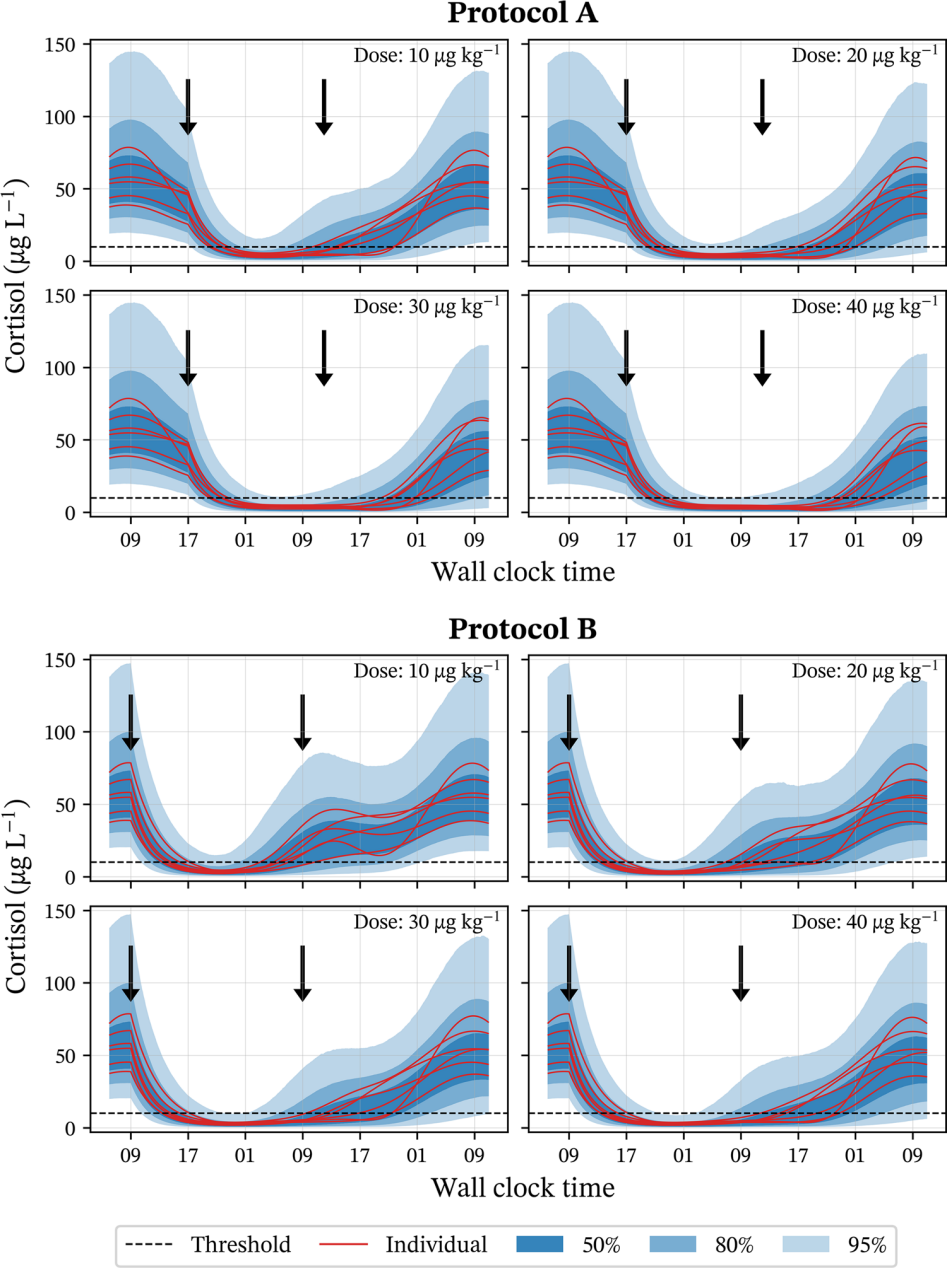
Simulated time courses including model-predicted variability for healthy horses undergoing the DST protocols A and B with the modification of varying the test dose of 10, 20, 30 and 40 μg kg^−1^ dexamethasone. The black dashed line represents the threshold of 10 μg kg^−1^ proposed by Dybdal et al. [1]. Arrows indicate time for drug administration at 9.00 o’clock (protocol B) or 17.00 o’clock (protocol A) and blood sample 19 h (protocol A) or 24 h (protocol B) after administration. Parameter uncertainty and IIV are incorporated into simulations. This variability is shown by blue shaded areas representing increasing amounts of variability (see legend). Predicted time courses for the horses in this study are shown as solid red lines (Color figure online)

We simulated time courses for healthy horses at four different dose levels (10, 20, 30 and 40 μg kg^−1^ dexamethasone administered intravenously) following protocols A and B. The resulting Fig. 4 shows predicted variability in possible time-courses resulting from IIV and parameter uncertainty. Additionally, predicted time courses under protocol A and B for the horses involved in this study are shown.

The variability in cortisol response around the threshold value (10 μg L^−1^) proposed in Dybdal et al. [1] was high after administration of 10 μg kg^−1^ but decreased steadily for increasing dose levels (Fig. 4). After administration of the 40 μg kg^−1^ dose variability in cortisol response 19 h after drug administration was lower than after the three lower doses. The model-predicted cortisol response shows that, even after the highest dose, individuals from a healthy population might have cortisol plasma concentrations greater than 10 μg L^−1^ at sampling time.

Systematic differences between the simulations of protocol A and B are shown in Fig. 4. Following protocol A, dexamethasone is administered at 17.00 o’clock, which coincides with cortisol decrease following the model-predicted circadian rhythm. Protocol B assumes administration at 9.00 o’clock which coincides with a peak in predicted cortisol concentration. As can also be seen from the individual predictions (Fig. 4), cortisol concentrations at administration time are generally lower following protocol A than B. At sampling time, 19 h (protocol A) or 24 h (protocol B) after administration, predicted concentrations for protocol A were consistently lower than for protocol B. However, differences grew smaller with increasing dexamethasone administration. For the highest dose (40 μg kg^−1^), remaining dexamethasone concentration in blood plasma was predicted to 309 ng L^−1^ (varying between 101 to 694 ng L^−1^) at sampling time for protocol A and 155 ng L^−1^ (varying between 34 to 391 ng L^−1^) at sampling time for protocol B. Suppressed cortisol concentration after administration of the largest dose of dexamethasone (40 μg kg^−1^) was lowest roughly between 5.00 o’clock and 12.00 o’clock for protocol A and 19.00 o’clock and 3.00 o’clock for protocol B (Fig. 4).

### Prediction of sensitivity and specificity for overnight DST protocols

Sensitivity and specificity were predicted through Monte Carlo simulations. All results are reported as median (95% CI). For protocol A, a sensitivity of 99.1% (93.1% to 99.7%) and a specificity of 83.4% (63.3% to 99.7%) were predicted. For protocol B, a sensitivity of 99.2% (94.2% to 99.8%) and a specificity of 77.8% (52.8% to 96.8%) were predicted. Sensitivities for both protocols turned out equal. Specificity tended towards lower values for protocol B but there is no substantial difference, given the spread of the credible intervals.

## Discussion

In this study, we used an NLME approach to investigate the IIV in dexamethasone exposure and cortisol response in horses. The aim was to use the adjusted model to scrutinize DST protocol designs. We analysed two proposed test protocols through simulation, found systematic differences in resulting cortisol time courses and predicted sensitivity and specificity for each protocol.

The PK/PD model used in this study was based on a previously published model (originally published in [26] and used in [12]). For the purpose of this work, some parts of the original model were simplified and re-parametrised. An analytical solution of the cortisol model equations (Eqs. 2–4) was used to initialise the parameter estimation (see Eq. 5). This was necessary since the cortisol response model does not have a constant steady state, which is typically used for initialisation. The calibrated model (Eqs. 1, 2) mimicked the overall tendency of the experimental data well. Individual PK parameter estimates (Table S1) and most PD parameters (Table S2) agree with previously reported values [12]. Parameter *α* was not directly comparable, since a different parametrisation of the model is used in this study. In comparison to Ekstrand et al. [12], the model in this study does not use a second delay compartment for the cortisol response. This affects individual estimates for parameter *k*_out_ which were systematically lower in this study, about half the previously estimated values. We found no evidence in the data to motivate the necessity of an additional delay compartment. Parameters *k*_avg_, *α* and *IC*_50_ showed evidence of non-zero IIV.

Large improvements in estimated precision for parameter *n* were achieved in this study. However, considerable uncertainty in some estimated parameters remained, notably *Cl*_*d*_, *α*, *t*_0_ and *IC*_50_. Dexamethasone exposure data (Figs. 2, S1) suggest a two-compartment model, but the influence of the second compartment is subtle, which explains the large uncertainty in parameter estimates of *Cl*_*d*_. Parameters *α* and *t*_0_ are mostly informed by baseline measurements, as their influence is diminished by dexamethasone administration. However, as reported in the results section (Figs. 2, S2) irregular fluctuations in baseline measurements confound the underlying oscillation of the cortisol response, explaining the challenge in precise estimation of these two parameters. Irregular fluctuations in baseline measurements can be explained by handling stress [38] as well as pulsatility in cortisol secretion [39].

The multiple sources of uncertainty, incorporation of IIV as well as the aim to utilize the calibrated model for prediction motivated our use of a Bayesian approach. The resulting posterior parameter distribution includes the general tendency of the estimated parameter values, as well as variability in form of IIV and parameter uncertainty. When IIV is included through the framework of NLME, parameter estimation can get more robust, since parameter estimates for one subject are informed by estimates of other subjects [22]. This can be seen in parameter *n*, which was estimated with much higher precision in this study than in Ekstrand et al. [12]. This holds for the Bayesian approach as well as the maximum likelihood approach. However, explicit inclusion of parameter uncertainty, when using a Bayesian approach, allows for straight forward model prediction, despite some remaining parameter uncertainty [25].

One clinically relevant application of modelling the cortisol response is evaluation of DST protocols. The overnight DST is commonly used when testing for PPID. Test protocols tend to be straight forward to be usable in both equine-clinics as well as in the field and typically involve only one or two samples. We investigated two overnight DST protocols through simulations, including an analysis of their sensitivity and specificity. Both protocols define a negative outcome as suppressed cortisol response to less than 10 μg L^−1^ in blood plasma 19 or 24 h after administration of 40 μg kg^−1^ dexamethasone [1, 11]. In our simulations dexamethasone plasma concentration at sampling time was was predicted to be 101–694 ng L^−1^ for protocol A and 34–391 ng L^−1^ for protocol B. This result is consistent with experimental data [16, 17]. Concentration at sampling time was above potency (22–54 ng L^−1^) for most individuals. However, there is a larger risk for dexamethasone concentration to fall below the potency value when following protocol B. Combined with the IIV in cortisol response, not all healthy horses will fall below the proposed threshold (Fig. 4).

All simulations were made under the assumption of intravenous administration of dexamethasone. Dybdal et al. [1] used intramuscular administration of dexamethasone. As shown by Soma et al. [17], dexamethasone plasma concentrations reach peak concentration after intramuscular administration with a slight time delay (about 15 min) compared to intravenous administration. However, concentrations at times 19 + h after administration, relevant for DST protocols, are comparable after intravenous as well as intramuscular administration. We therefore decided that it is reasonable to compare our simulations with the data published by Dybdal et al. [1].

For the purposes of model estimation only data from healthy horses was available. To simulate sensitivity for DST protocols A and B, we hypothesized that sick horses do not respond to dexamethasone administration. Their responses were therefore simulated at baseline. This was motivated by the known mechanism that cortisol suppression through dexamethasone is challenged in horses affected by PPID [6]. PPID also seems to lead to a reduction in circadian rhythm and an increase in average baseline concentrations [1]. However, numerical values quantifying these latter differences for the race of horses used in this study were not available. Incorporating these changes into the hypothesis stated above would have increased the predicted sensitivity, which was close to 100% already. To the best of our knowledge, there is no finding about differences in dexamethasone kinetics between healthy horses and those affected by PPID.

Sensitivity and specificity were determined through simulation for both protocols. The predicted specificity for protocol A, Dybdal et al. [1] reporting 100% specificity, was 63.3% to 99.7% and for protocol B, Frank et al. [11] reporting 76% specificity, it was 52.8% to 96.8%. The consistency between experimentally determined results and our simulations makes us confident that our model captured the overall cortisol response in healthy horses well. Predicted sensitivity for protocol A, Dybdal et al. [1] reporting 100% sensitivity, was 93.1% to 99.7% and for protocol B, Frank et al. [11] reporting 65% sensitivity, it was 94.2% to 99.8%. An explanation for the large discrepancy between our simulation and the experimental result for protocol B might be that our hypothesis only applies to a population of horses with clinically advanced PPID [40]. Such a population was considered by Dybdal et al. [1] and simulated sensitivity for protocol A is in agreement with experimental results. Future work might consider simulations of horses in which maximum suppression is decreased, imitating a mechanism that still reacts to dexamethasone suppression but at a reduced intensity.

DST protocols A and B use different starting times (afternoon vs. morning) and different waiting times until cortisol sampling (19 h vs. 24 h). Simulations show that it can be more effective to administer dexamethasone during a descending phase in cortisol’s circadian rhythm (Fig. 4). Also, cortisol’s circadian rhythm can interfere with the effectiveness of dexamethasone suppression (Fig. 4, protocol B, upswing after suppression coincides with natural upswing in cortisol production). However, no substantial difference in test outcome between protocols A and B was found in this study. A finding also consistent with a study by Sojka et al. [41] that investigated the influence of starting time on the DST and found no statistically significant difference between starting in the morning or the afternoon.

Simulations showed that a dose of 40 μg kg^−1^ dexamethasone is necessary to conduct the current test protocols, with lower doses leading to an increased number of false positives (Fig. 4). Small changes in dose around the potency value result in large reduction of cortisol average baseline and amplitude (Fig. 3), but this diminishes quickly for concentrations above the potency value. This has implications on dose selection and sampling time points in the DST. A dose-increment and consequently increased plasma exposure will decrease the number of false positive test results in healthy individuals. It is important to consider that negative adverse effects from glucocorticoids, e.g., hyperglycaemia, hyperinsulinemia and possibly laminitis, are assumed to increase with higher doses [42–44]. Shorter waiting time until sampling could further decrease the number of false positives. Cortisol is suppressed quickly by dexamethasone and peak suppression is reached earlier than 19 + h after administration (Fig. 4). However, this could mean sampling in the late evening or at night (between 19.00 o’clock to 3.00 o’clock for protocol B), which might be unacceptable in the field. Protocol A has better potential for improvement since suppression is at its maximum during early morning hours (between 5.00 o’clock to 12.00 o’clock for protocol A). Existing protocols show that practical applicability is an important factor in their design.

Ideally, serial sampling would be used. An extended sampling protocol with 2–3 additional samples would provide more information about cortisol behaviour and more reliable test results. Additional collection of an unaffected cortisol baseline would allow estimation of the typical cortisol response for the particular horse and allow for an increase in the predictive power of the test. However, this is not a suitable strategy for clinical routine, especially not in the field and the cost would also be accepted in lower extent by the owners. Given the challenge of producing a protocol that clinicians can perform, 2–3 samples after drug administration is the maximum to be collected. Therefore, some uncertainty in interpretation of test results remains and the diagnosis must be based not only on the test outcome.

## Conclusion

Our study presents an improved model structure and parameter estimates for cortisol concentration in horses during intervention with dexamethasone. The use of non-linear mixed effects modelling allowed estimation of variation between individuals finding IIV in parameters *k*_avg_, *α* and *IC*_50_. Using a Bayesian approach allowed straight-forward propagation of uncertainty to simulations. The adjusted model was successfully used to scrutinize clinical test protocols through simulation. The model output and simulations indicated the importance of dose selection with doses below 40 μg kg^−1^ performing unfavourably. Sampling time was also found to be of importance and simulations showed that waiting times in the window 10 to 17 h could improve test performance. In addition, it was found that administrating dexamethasone in synchronisation with the down-swing in cortisol’s circadian rhythm can allow for a slight prolongation in waiting time.

### Electronic supplementary material

Below is the link to the electronic supplementary material.

Supplementary material 1 (DOCX 7139 kb)

## Appendix

### Cortisol concentration for constant dexamethasone concentration

The solution to the cortisol model equations (Eqs. 2–4) can be found analytically for constant dexamethasone concentration *C*_*p*_ = *C*_*p*, eq_.

Mathematically, Eq. 2 describes a first-order differential equation driven by an external oscillation (the turnover rate). Using methods described by Farkas [45], it can be shown that there exists a unique oscillating solution to Eq. 2. This solution will have the same period as the driving turnover rate and any other solution will tend towards this oscillating solution. Assume that the model equations are solved by

13 $$R_{eq} (t) = a + b \cdot \cos (\omega\, (t - t_{0} )) + c \cdot \sin (\omega\, (t - t_{0} ))\,,$$

where *ω* = 2*π*/24 h^−1^ and *t*_0_ is the phase shift in the turnover rate (Eq. 3). Solving for *a*, *b* and *c* gives

14 $$\begin{aligned} 
a & = \frac{{k_{\text{avg}} }}{{k_{\text{out}} }} \cdot \left( {1 - \frac{{I_{\text{max} } C_{{p,\,{\text{eq}}}}^{n} }}{{IC_{50}^{n} + C_{{p,\,{\text{eq}}}}^{n} }}} \right)\,, \\ 
b & = \frac{{k_{\text{avg}} \cdot \alpha \cdot k_{\text{out}} }}{{k_{\text{out}}^{2} + \omega^{2} }} \cdot \left( {1 - \frac{{I_{\text{max} }\,C_{{p,\,{\text{eq}}}}^{n} }}{{IC_{50}^{n} + C_{{p,\,{\text{eq}}}}^{n} }}} \right)\,, \\ 
c & = \frac{{k_{\text{avg}} \cdot \alpha \cdot \omega }}{{k_{\text{out}}^{2} + \omega^{2} }} \cdot \left( {1 - \frac{{I_{\text{max} }\, C_{p,\,\text{eq}}^{n} }}{{IC_{50}^{n} + C_{{p,\,{\text{eq}}}}^{n} }}} \right). \\ \end{aligned}$$

To determine characteristic parameters for the equilibrium oscillation, it is more convenient to write Eq. 13 in the form $R_{eq} (t) = A + B\cos (\omega\,(t - C))$ where *A*, *B* and *C* are as in Eq. 6. This representation can be found by standard arguments for the sum of trigonometric functions. It can be seen from these equations that *A* and *B* depend on the drug concentration, while *C* does not.

### Determination of prior parameters

Prior beliefs about means and variances for hierarchically distributed parameters were quantified through a meta-analysis of Ekstrand et al. [12]. Therein, point estimates of parameter values and their relative precision (coefficient of variation, CV) in percent were reported. If *θ* is the reported point estimate and *CV* its relative precision then standard errors *σ* were calculated as *σ* = (*CV*·*θ*)/100. To arrive at an estimate for the population mean and population variance for a parameter, e.g., *Cl*, weighted averages of the point estimates were formed. Each estimate was weighted by its squared standard error. Let *θ*_*i*_ and *σ*_*i*_ be the estimate and standard error determined for the *i*th horse. Then

15 $$\hat{\mu } = \frac{{\mathop \sum \nolimits_{i = 1}^{6} \frac{{\theta_{i} }}{{\sigma_{i}^{2} }}}}{{\mathop \sum \nolimits_{i = 1}^{6} \frac{1}{{\sigma_{i}^{2} }}}}\;{\text{and}}\;\hat{\omega }^{2} = \left( {\frac{{\mathop \sum \nolimits_{i = 1}^{6} \frac{{\theta_{i}^{2} }}{{\sigma_{i}^{2} }}}}{{\mathop \sum \nolimits_{i = 1}^{6} \frac{1}{{\sigma^{2} }}}}} \right) - \hat{\mu }^{2} ,$$

where $\hat{\mu }$ and $\hat{\omega }^{2}$ are the population mean and variance, respectively.

Parameters were modelled hierarchically either through a log-normal distribution (e.g., *Cl*), a (scaled) logit-normal distribution (for *α*, *t*_0_ and *I*_max_) or a normal distribution (for *n*). For all parameters the prior location parameters *m*_0_ and *s*_0_ were determined by matching mean and variance of the respective distribution with those in Eq. 15.

For a normal distribution $\hat{\mu }$ and $\hat{\omega }^{2}$ can be used directly. Analytical formulas for the mean and variance of the log-normal distribution exist. It holds that

16 $$\mu = \exp \left( {m + \frac{{s^{2} }}{2}} \right)\;{\text{and}}\;\omega^{2} = \left( {\exp \left( {s^{2} } \right) - 1} \right) \cdot \exp \left( {2m + s^{2} } \right),$$

where *m* and *s* are the location and scale parameters of the log-normal distribution. *μ* and *ω*^2^ were matched with $\hat{\mu }$ and $\hat{\omega }^{2}$ in Eq. 15 to determine *m* and *s*. Values can be found in the table below.

In case of the (scaled) logit-normal distribution no closed-form solutions for mean and variance exist. Therefore, moments were calculated by numerical integration using SciPy’s fixed_quad function [46]. Numerical optimization was used to determine location and scale parameters. The difference between numerically approximated moments and those in Eq. 15 was used as a target function. SciPy’s fsolve, which implements methods from MINPACK [47], was used for numerical optimization. The resulting optimized parameters were *m* and *s*. Values can be found in the table below.

The parameter controlling the average turnover rate *k*_avg_ was not directly given by Ekstrand et al. [12]. Instead, the parameter *R*_*ij*_ = *k*_avg, *ij*_/*k*_out, *i*_ for individual *i* and occasion *j* was given.^1^ Note that the parameter *k*_avg_ was allowed to vary by experimental occasion in Ekstrand et al. [12]. In the model presented herein, the parameter *k*_avg_ was modelled with IIV instead. Individual- and occasion-specific parameter estimates for *k*_avg, *ij*_ could easily be recovered as *k*_avg, *ij*_ = *R*_*ij*_·*k*_out, *i*_. Relative precisions were used to calculate the standard errors $\sigma_{{R_{ij} }}$ and $\sigma_{{k_{{{\text{out}},i}} }}$ of each individual-/occasion-dependent estimate. Standard errors for *k*_avg, *ij*_ were then approximated by propagation of uncertainty [48]

17 $$\sigma_{{k_{{{\text{avg}},ij}} }} \approx \left| {k_{{{\text{avg}},ij}} } \right|\sqrt {\left( {\frac{{\sigma_{{R_{ij} }} }}{{R_{ij} }}} \right)^{2} + \left( {\frac{{\sigma_{{k_{{{\text{out}},i}} }} }}{{k_{{{\text{out}},i}} }}} \right)^{2} } .$$

Summing over all individuals and occasions, weighted averages, as in Eq. 15, were formed. These were matched with mean and variance of a log-normal distribution to get location *m* and scale *s* for *k*_*avg*_. Values can be found in the table below.

Location parameters *m* and *s* for prior distribution of hyper-parameters *μ* and *τ*.

**Table:**

Parameter *θ*	*Cl*	*Cl*_*d*_	*V*_*c*_	*V*_*t*_	*k*_avg_	*α*	*t*_0_	*k*_out_	*I*_max_	*IC*_50_	*n*
*m*_0_	− 0.71	− 1.7	− 0.21	− 0.15	3.0	− 0.82	− 0.71	− 0.56	1.6	− 4.1	0.75
*s*_0_	0.064	0.46	0.27	0.16	0.46	0.98	0.10	0.26	0.34	0.73	0.36

### Calculation of simulation of sensitivity and specificity

By sampling *j* = 1,…,* M* individual parameter vectors, called ${\varvec{\uptheta}}_{j}^{i}$ for the *i*th set of hyper-parameters, sensitivity *Se*^(*i*)^ and specificity *Sp*^(*i*)^ can be calculated as

18 $$Se^{(i)} \approx \frac{1}{M}\mathop \sum \limits_{j = 1}^{M} \left[ {1 - \varPhi \left( {\frac{{10 - \hat{R}({\varvec{\uptheta}}_{j}^{(i)} )}}{{\sqrt {\hat{R}({\varvec{\uptheta}}_{j}^{(i)} )^{2} \sigma_{1}^{2} + \sigma_{2}^{2} } }}} \right)} \right]$$

and

19 $$Sp^{(i)} \approx \frac{1}{M}\mathop \sum \limits_{j = 1}^{M} \left[ {\varPhi \left( {\frac{{10 - \hat{R}({\varvec{\uptheta}}_{j}^{(i)} )}}{{\sqrt {\hat{R}({\varvec{\uptheta}}_{j}^{(i)} )^{2} \sigma_{1}^{2} + \sigma_{2}^{2} } }}} \right) + \varPhi \left( {\frac{{\hat{R}({\varvec{\uptheta}}_{j}^{(i)} )}}{{\sqrt {\hat{R}({\varvec{\uptheta}}_{j}^{(i)} )^{2} \sigma_{1}^{2} + \sigma_{2}^{2} } }}} \right) - 1} \right]\,.$$

Here, *Φ* is the cumulative distribution function of the standard normal distribution, and $\hat{R}({\varvec{\uptheta}}_{j}^{(i)} )$ is the predicted cortisol measurement obtained in the DST. In the case of sensitivity, sick subjects were simulated and $\hat{R}({\varvec{\uptheta}}_{j}^{(i)} )$ was therefore predicted at baseline without drug. For specificity, healthy subjects were simulated and $\hat{R}({\varvec{\uptheta}}_{j}^{(i)} )$ was predicted after administration of 40 μg kg^−1^ dexamethasone. Repeating this procedure for many sets of hyper-parameters and a reasonably high value of *M* leads to a distribution for sensitivity and specificity, which can be summarised by median and credible interval. A derivation of Eqs. 18, 19 is given in the Supplementary.

## References

[1] Dybdal NO, Hargreaves KM, Madigan JE, Gribble DH, Kennedy PC, Stabenfeldt GH (1994). Diagnostic testing for pituitary pars intermedia dysfunction in horses. J Am Vet Med Assoc.

[2] de Grauw JC, Visser-Meijer MC, Lashley F, Meeus P, van Weeren PR (2016). Intra-articular treatment with triamcinolone compared with triamcinolone with hyaluronate: a randomised open-label multicentre clinical trial in 80 lame horses. Equine Vet J.

[3] Leclere M, Lefebvre-Lavoie J, Beauchamp G, Lavoie JP (2010). Efficacy of oral prednisolone and dexamethasone in horses with recurrent airway obstruction in the presence of continuous antigen exposure. Equine Vet J.

[4] MacHarg MA, Bottoms GD, Carter GK, Johnson MA (1985). Effects of multiple intramuscular injections and doses of dexamethasone on plasma cortisol concentrations and adrenal responses to acth in horses. Am J Vet Res.

[5] Eiler H, Oliver J, Goble D (1979). Adrenal gland function in the horse: effect of dexamethasone on hydrocortisone secretion and blood cellularity and plasma electrolyte concentrations. Am J Vet Res.

[6] Orth DN, Holscher MA, Wilson MG, Nicholson WE, Plue RE, Mount CD (1982). Equine cushing’s disease: plasma immunoreactive proopiolipomelanocortin peptide and cortisol levels basally and in response to diagnostic tests. Endocrinol.

[7] McFarlane D, Dybdal N, Donaldson MT, Miller L, Cribb AE (2005). Nitration and increased alpha-synuclein expression associated with dopaminergic neurodegeneration in equine pituitary pars intermedia dysfunction. J Neuroendocrinol.

[8] McFarlane D (2007). Advantages and limitations of the equine disease, pituitary pars intermedia dysfunction as a model of spontaneous dopaminergic neurodegenerative disease. Ageing Res Rev.

[9] Ireland JL, McGowah CM (2018). Epidemiology of pituitary pars intermedia dysfunction: a systematic literature review of clinical presentation, disease prevalence and risk factors. Vet J.

[10] McGowan TW, Pinchbeck GP, McGowan CM (2013). Prevalence, risk factors and clinical signs predictive for equine pituitary pars intermedia dysfunction in aged horses: prevalence and risk factors for equine PPID. Equine Vet J.

[11] Frank N, Andrews FM, Sommardahl CS, Eiler H, Rohrbach BW, Donnell RL (2006). Evaluation of the combined dexamethasone suppression/thyrotropin-releasing hormone stimulation test for detection of pars intermedia pituitary adenomas in horses. J Vet Intern Med.

[12] Ekstrand C, Ingvast-Larsson C, Olsen L, Hedeland M, Bondesson U, Gabrielsson J (2016). A quantitative approach to analysing cortisol response in the horse. J Vet Pharmacol Ther.

[13] Toutain PL, Oukessou M, Autefage A, Alvinerie M (1988). Diurnal and episodic variations of plasma hydrocortisone concentrations in horses. Domest Anim Endocrinol.

[14] Hart KA, Dirikolu L, Ferguson C, Norton NA, Barton MH (2010). Daily endogenous cortisol production and hydrocortisone pharmacokinetics in adult horses and neonatal foals. Am J Vet Res.

[15] Gabrielsson J, Weiner D (2016). Pharmacokinetic & pharmacodynamic data analysis: concepts and applications.

[16] Soma LR, Uboh CE, Luo Y, Guan F, Moate PJ, Boston RC (2005). Pharmacokinetics of dexamethasone with pharmacokinetic/pharmacodynamic model of the effect of dexamethasone on endogenous hydrocortisone and cortisone in the horse. J Vet Pharmacol Ther.

[17] Soma LR, Uboh CE, Liu Y, Li X, Robinson MA, Boston RC, Colahan PT (2013). Pharmacokinetics of dexamethasone following intra-articular, intravenous, intramuscular, and oral administration in horses and its effects on endogenous hydrocortisone. J Vet Pharmacol Ther.

[18] Toutain PL, Brandon RA, de Pomyers H, Alvinerie M, Baggot JD (1984). Dexamethasone and prednisolone in the horse: pharmacokinetics and action on the adrenal gland. Am J Vet Res.

[19] Cunningham FE, Rogers S, Fischer JH, Jensen RC (1996). The pharmacokinetics of dexamethasone in the thoroughbred racehorse. J Vet Pharmacol Ther.

[20] Grady JA, Davis EG, KuKanich B, Sherck AB (2010). Pharmacokinetics and pharmacodynamics of dexamethasone after oral administration in apparently healthy horses. Am J Vet Res.

[21] Ekstrand C, Bondesson U, Gabrielsson J, Hedeland M, Kallings P, Olsén L, Ingvast-Larsson C (2015). Plasma concentration-dependent suppression of endogenous hydrocortisone in the horse after intramuscular administration of dexamethasone-21-isonicotinate. J Vet Pharmacol Ther.

[22] Ette EI, Williams PJ (2007). Pharmacometrics: the science of quantitative pharmacology.

[23] Davidian M, Giltinan DM (1995). Nonlinear models for repeated measurement data.

[24] Bon C, Toutain PL, Concordet D, Gehring R, Martin-Jimenez T, Smith J, Pelligand L, Martinez M, Whittem T, Riviere JE, Mochel JP (2018). Mathematical modeling and simulation in animal health. Part III: using nonlinear mixed-effects to characterize and quantify variability in drug pharmacokinetics. J Vet Pharmacol Ther.

[25] Gelman A, Carlin JB, Stern HS, Dunson DB, Vehtari A, Rubin DB (2013). Bayesian data analysis.

[26] Chakraborty A, Krzyzanski W, Jusko WJ (1999). Mathematical modeling of circadian cortisol concentrations using indirect response models: comparison of several methods. J Pharmacokinet Pharmacodyn.

[27] Krzyzanski W, Chakraborty A, Jusko WJ (2000). Algorithm for application of fourier analysis for biorythmic baselines of pharmacodynamic indirect response models. Chronobiol Int.

[28] Lewandowski D, Kurowicka D, Joe H (2009). Generating random correlation matrices based on vines and extended onion method. J Multivar Anal.

[29] Gelman A (2006). Prior distributions for variance parameters in hierarchical models. Bayesian Anal.

[30] Parikh R, Mathai A, Parikh S, Sekhar GC, Thomas R (2008). Understanding and using sensitivity, specificity and predictive values. Indian J Ophthalmol.

[31] Carpenter B, Gelman A, Hoffman MD, Lee D, Goodrich B, Betancourt M, Brubaker M, Guo J, Li P, Riddell A (2017). Stan: a probabilistic programming language. J Stat Softw.

[32] Hoffman MD, Gelman A (2014). The no-u-turn sampler: adaptively setting path lengths in Hamiltonian Monte Carlo. J Mach Learn Res.

[33] Neal RM, Brooks S, Gelman A, Jones GL, Meng X-L (2011). MCMC using Hamiltonian dynamics. Handbook of Markov chain Monte Carlo.

[34] Betancourt M, Girolami M (2013) Hamiltonian Monte Carlo for hierarchical models. http://arxiv.org/abs/1312.0906. Accessed 29 Aug 2018

[35] Gelman A, Rubin DB (1992). Inference from iterative simulation using multiple sequences. Stat Sci.

[36] Betancourt M (2018) A conceptual introduction to Hamiltonian Monte Carlo. https://arxiv.org/pdf/1701.02434.pdf. Accessed 29 Aug 2018

[37] Comets E, Brendel K, Mentré F (2010). Model evaluation in nonlinear mixed effect models, with applications to pharmacokinetics. J Soc Fr Stat.

[38] Irvine CH, Alexander SL (1994). Factors affecting the circadian rhythm in plasma cortisol concentrations in the horse. Domest Anim Endocrinol.

[39] Toutain PL, Laurentie M, Autefage A, Alvinerie M (1988). Hydrocortisone secretion: production rate and pulse characterization by numerical deconvolution. Am J Physiol.

[40] Spelta C (2015). Equine pituitary pars intermedia dysfunction: current perspectives on diagnosis and management. Int J Vet Med.

[41] Sojka JE, Johnson MA, Bottoms GD (1993). The effect of starting time on dexamethasone suppression test results in horses. Domest Anim Endocrinol.

[42] Tiley HA, Geor RJ, McCutcheon LJ (2007). Effects of dexamethasone on glucose dynamics and insulin sensitivity in healthy horses. Am J Vet Res.

[43] Welsh CE, Duz M, Parkin TDH, Marshall JF (2017). Disease and pharmacologic risk factors for first and subsequent episodes of equine laminitis: a cohort study of free-text electronic medical records. Prev Vet Med.

[44] Bailey SR (2010). Corticosteroid-associated laminitis. Vet Clin N Am Equine Pract.

[45] Farkas M (1994). Periodic motions.

[46] Jones E, Oliphant T, Peterson P et al (2018) SciPy: open source scientific tools for Python. http://www.scipy.org/. Accessed 29 Aug 2018

[47] More JJ, Garbox BS, Hillstrom KE (1980) User guide for MINPACK-1. Argonne National Laboratory. http://cds.cern.ch/record/126569?ln=en. Accessed 29 Aug 2018

[48] Taylor JR (1997). An introduction to error analysis: the study of uncertainties in physical measurements.

