# Supplementary material for: Modelling of oscillatory cortisol response in horses using a Bayesian population approach for evaluation of dexamethasone suppression test protocols
Source: J Pharmacokinet Pharmacodyn. 2019 Jan 23;46(1):75–87. doi: 10.1007/s10928-018-09617-0 (PMC6394511; doi:10.1007/s10928-018-09617-0)
Supplement: Supplementary file 1 — Supplementary material 1 (DOCX 7139 kb) [file 10928_2018_9617_MOESM1_ESM.docx]

Supplementary for: “Modelling of oscillatory cortisol response in horses using a Bayesian population approach for evaluation of dexamethasone suppression test protocols”

## Authors

Felix Held (1,2,4), Carl Ekstrand (3), Marija Cvijovic (2), Johan Gabrielsson (3), and Mats Jirstrand (1)

## Affiliations

1. Fraunhofer-Chalmers Centre, Chalmers Science Park, Gothenburg, Sweden
2. Department of Mathematical Sciences, Chalmers University of Technology and University of Gothenburg, Gothenburg, Sweden
3. Department of Biomedical Sciences and Veterinary Public Health, Swedish University of Agricultural Sciences, Uppsala, Sweden
4. Address correspondence to felix.held@chalmers.se

# Supplementary to Methods and Materials

## Derivation of formulas for model-derived sensitivity and specificity

Sensitivity and specificity are population-dependent quantities. In the context of the model this implies that each set of hyper-parameters results in one realisation of a sensitivity and a specificity value.

Let *T*_DST_ be the threshold for the overnight DST, *S* the true disease state of the subject (*S* = *H* for healthy and *S* = *D* for diseased), **θ** a realisation of the individual parameters and **σ** the corresponding residual variance parameters. Denote the cortisol concentration measured during the DST by *R*_DST_. Let *T*(*R*_DST_) be the test outcome. Then

$T(R_{\mathrm{DST}})=\left\{ \begin{matrix} 0 & R_{\mathrm{DST}}\leq T_{\mathrm{DST}} \\ 1 & R_{\mathrm{DST}}>T_{\mathrm{DST}} \end{matrix} \right.$ (1)

A generalisation of the measurement equation for cortisol (Eq. 8 in main text) can be written as

$R_{\mathrm{DST}}=\hat{R}(\boldsymbol{\theta},S)+g(\hat{R}(\boldsymbol{\theta},S),\boldsymbol{\sigma})\cdot\varepsilon, R_{\mathrm{DST}}\geq0$ (2)

where *R̂*(**θ**, *S*) is the model-predicted cortisol concentration dependent on **θ** and *S*, *g* is a function describing the residual error variance model and *ε* has a standard normal distribution.

The probability of the test giving a positive outcome for a subject having the disease is

$\begin{matrix} P(T(R_{\mathrm{DST}})=1|\boldsymbol{\theta},\boldsymbol{\sigma},S=D) & =P(\hat{R}(\boldsymbol{\theta},D)+g(\hat{R}(\boldsymbol{\theta},D),\boldsymbol{\sigma})\cdot\varepsilon>T_{\mathrm{DST}} | \boldsymbol{\theta},\boldsymbol{\sigma})= \\ & =1-P\left( \varepsilon\leq\frac{T_{\mathrm{DST}}-\hat{R}(\boldsymbol{\theta},D)}{g(\hat{R}(\boldsymbol{\theta},D),\boldsymbol{\sigma})} \right)= \\ & =1-\Phi\left( \frac{T_{\mathrm{DST}}-\hat{R}(\boldsymbol{\theta},D)}{g(\hat{R}(\boldsymbol{\theta},D),\sigma)} \right) \end{matrix}$ (3)

where *Φ* is the cumulative distribution function of the standard normal distribution. The probability of the test giving a negative outcome for a healthy subject is

$\begin{matrix} P(T(R_{\mathrm{DST}})=0|\boldsymbol{\theta},\boldsymbol{\sigma},S=H) & =P(0\leq\hat{R}(\boldsymbol{\theta},H)+g(\hat{R}(\boldsymbol{\theta},H),\boldsymbol{\sigma})\cdot\varepsilon\leq T_{\mathrm{DST}})= \\ & =P\left( -\frac{\hat{R}(\boldsymbol{\theta},H)}{g(\hat{R}(\boldsymbol{\theta},H),\boldsymbol{\sigma})}\leq\varepsilon\leq\frac{T_{\mathrm{DST}}-\hat{R}(\boldsymbol{\theta},H)}{g(\hat{R}(\boldsymbol{\theta},H),\boldsymbol{\sigma})} \right)= \\ & =\Phi\left( \frac{T_{\mathrm{DST}}-\hat{R}(\boldsymbol{\theta},H)}{g(\hat{R}(\boldsymbol{\theta},H),\boldsymbol{\sigma})} \right)-\Phi\left( -\frac{\hat{R}(\boldsymbol{\theta},H)}{g(\hat{R}(\boldsymbol{\theta},H),\boldsymbol{\sigma})} \right) \\ & =\Phi\left( \frac{T_{\mathrm{DST}}-\hat{R}(\boldsymbol{\theta},H)}{g(\hat{R}(\boldsymbol{\theta},H),\boldsymbol{\sigma})} \right)+\Phi\left( \frac{\hat{R}(\boldsymbol{\theta},H)}{g(\hat{R}(\boldsymbol{\theta},H),\boldsymbol{\sigma})} \right)-1 \end{matrix}$ (4)

Denote all collected experimental data by **Y** and the full set of hyper-parameters for all parameters by **Ψ** (mean values *μ*, standard deviations *τ* and correlations). For the posterior distribution of sensitivity *Se* it holds that

$\begin{matrix} p(Se|\mathbf{Y}) & =\int P(T(R_{\mathrm{DST}})=1|\boldsymbol{\theta},S=D)\cdot p(\boldsymbol{\theta},\boldsymbol{\sigma}|\mathbf{Y}) d(\boldsymbol{\theta},\boldsymbol{\sigma})= \\ & =\int P(T(R_{\mathrm{DST}})=1|\boldsymbol{\theta},S=D)\int p(\boldsymbol{\theta},\boldsymbol{\sigma}|\boldsymbol{\Psi},\mathbf{Y})\cdot p(\boldsymbol{\Psi}|\mathbf{Y}) d(\boldsymbol{\Psi}) d(\boldsymbol{\theta},\boldsymbol{\sigma})= \\ & =\int\int\left[ 1-\Phi\left( \frac{T_{\mathrm{DST}}-\hat{R}(\boldsymbol{\theta},S)}{g(\hat{R}(\boldsymbol{\theta},S),\boldsymbol{\sigma})} \right) \right]\cdot p(\boldsymbol{\theta}|\boldsymbol{\Psi}) d\boldsymbol{\theta} p(\boldsymbol{\Psi},\boldsymbol{\sigma}|\mathbf{Y}) d(\boldsymbol{\Psi},\boldsymbol{\sigma}) \end{matrix}$ (5)

where we have used that

$p(\boldsymbol{\theta},\boldsymbol{\sigma}|\boldsymbol{\Psi},\mathbf{Y})\cdot p(\boldsymbol{\Psi}|\mathbf{Y})=\frac{p(\boldsymbol{\theta},\boldsymbol{\sigma},\boldsymbol{\Psi}|\mathbf{Y})}{p(\boldsymbol{\Psi}|\mathbf{Y})}\cdot p(\boldsymbol{\Psi}|\mathbf{Y})=p(\boldsymbol{\theta}|\boldsymbol{\Psi})\cdot p(\boldsymbol{\Psi},\boldsymbol{\sigma}|\mathbf{Y}).$ (6)

The last equality holds since **θ** does not depend on **σ**, and **θ** is conditionally independent of **Y** if **Ψ** is given. For the posterior distribution of specificity *Sp* it holds with similar arguments that

$p(Sp|\mathbf{Y})=\int\int\left[ \Phi\left( \frac{T_{\mathrm{DST}}-\hat{R}(\boldsymbol{\theta},H)}{g(\hat{R}(\boldsymbol{\theta},H),\boldsymbol{\sigma})} \right)+\Phi\left( \frac{\hat{R}(\boldsymbol{\theta},H)}{g(\hat{R}(\boldsymbol{\theta},H),\boldsymbol{\sigma})} \right)-1 \right]\cdot p(\boldsymbol{\theta}|\boldsymbol{\Psi}) d\boldsymbol{\theta} p(\boldsymbol{\Psi},\boldsymbol{\sigma}|\mathbf{Y}) d(\boldsymbol{\Psi},\boldsymbol{\sigma})$ (7)

Both integrals in Eqns. 5, 7 cannot be solved analytically and the densities for *Se* and *Sp* are therefore not directly available. Using the posterior distribution for the parameter estimates, samples from these distributions can be gained by following the procedure described in the appendix of the main text.

## Predicted population range

The expected range of parameters in the population was calculated after parameter estimation. For the parameters in the PK/PD model **θ** with hyperparameters **Ψ** the expected range of each parameter corresponds to the range of the respective marginal posterior predictive distribution. The overall posterior predictive distribution is

$p(\boldsymbol{\theta}|\mathbf{Y})=\int p(\boldsymbol{\theta}|\boldsymbol{\Psi})p(\boldsymbol{\Psi}|\mathbf{Y}) d\boldsymbol{\Psi}$ (8)

The samples for **Ψ** from the parameter posterior distribution can be used to approximate the integral

$p(\boldsymbol{\theta}|\mathbf{Y})\approx\frac{1}{N}\sum_{i=1}^{N} p(\boldsymbol{\theta}|\boldsymbol{\Psi}^{i})$ (9)

Samples from this approximative distribution can be easily obtained, since the analytical form of the distributions *p*(**θ**|**Ψ***^i^*) is known from the section “Statistical parameter model” and the approximation is a mixture distribution. It is enough to sample *i* from {1, …, *N*} and then sample $\hat{\boldsymbol{\theta}}$ from *p*(**θ**|**Ψ***^i^*) to obtain approximate samples from *p*(**θ**|**Y**). Marginals for the separate parameters are easily obtained by only considering a specific component of $\hat{\boldsymbol{\theta}}$.

# Supplementary results

## Estimated parameter values

Parameter estimates for individual horses are presented in Tables S1 and S2. These complement the reported population parameter values in Tables 1 and 2 in the main text.

**Table S1** Estimated parameter values for the drug exposure model per horse

| Parameter (Unit) | Horse | | | | | |
| --- | --- | --- | --- | --- | --- | --- |
|  | **1** | **2** | **3** | **4** | **5** | **6** |
| *Cl* (L h^-1^ kg^-1^) | 0.52 (0.46, 0.63) | 0.49 (0.42, 0.55) | 0.50 (0.43, 0.57) | 0.51 (0.45, 0.59) | 0.47 (0.39, 0.54) | 0.49 (0.43, 0.55) |
| *Cl_d_* (L h^-1^ kg^-1^) | 0.20 (0.082, 0.49) | 0.18 (0.062, 0.52) | 0.18 (0.079, 0.43) | 0.15 (0.058, 0.40) | 0.16 (0.072, 0.41) | 0.12 (0.051, 0.28) |
| *V_c_* (L kg^-1^) | 1.3 (0.65, 2.2) | 1.3 (0.73, 1.8) | 1.2 (0.75, 1.7) | 1.2 (0.66, 1.6) | 1.3 (0.77, 1.7) | 1.2 (0.77, 1.7) |
| *V_t_* (L kg^-1^) | 0.80 (0.49, 1.2) | 0.74 (0.45, 1.1) | 0.78 (0.51, 1.2) | 0.71 (0.46, 1.1) | 0.77 (0.49, 1.1) | 0.77 (0.49, 1.3) |

Values reported as median and 95 % credible interval

**Table S2** Estimated parameter values for the cortisol turnover model per horse

| Parameter (Unit) |  |  |  | Horse |  |  |
| --- | --- | --- | --- | --- | --- | --- |
|  | **1** | **2** | **3** | **4** | **5** | **6** |
| *k*_avg_ (μg L^-1^ h^-1^) | 13. (11., 17.) | 14. (11., 18.) | 19. (14., 26.) | 18. (13., 25.) | 10. (7.7, 15.) | 18. (13., 29.) |
| *α* (unitless) | 0.81 (0.62, 0.93) | 0.26 (0.084, 0.43) | 0.11 (0.021, 0.26) | 0.29 (0.13, 0.44) | 0.34 (0.15, 0.49) | 0.19 (0.060, 0.33) |
| *t*_0_ (h) | -3.4 (-4.3, -2.2) | -2.9 (-4.4, -0.69) | -2.8 (-4.7, 0.16) | -2.8 (-4.3, -0.81) | -3.2 (-4.8, -1.4) | -2.8 (-4.6, -0.27) |
| *k*_out_ (h^-1^) | 0.26 (0.23, 0.33) | 0.38 (0.31, 0.49) | 0.38 (0.30, 0.51) | 0.33 (0.26, 0.43) | 0.34 (0.26, 0.47) | 0.37 (0.28, 0.55) |
| *I*_max_ (unitless) | 0.96 (0.89, 0.99) | 0.95 (0.91, 0.99) | 0.95 (0.87, 0.99) | 0.94 (0.83, 0.98) | 0.93 (0.80, 0.98) | 0.94 (0.84, 0.98) |
| *IC*_50_ (ng L^-1^) | 26. (18., 38.) | 57. (40., 85.) | 41. (31., 58.) | 47. (32., 72.) | 29. (17., 48.) | 35. (24., 56.) |
| *n* (unitless) | 2.4 (1.5, 3.9) | 2.6 (1.7, 4.3) | 2.5 (1.6, 3.8) | 2.4 (1.4, 3.7) | 2.3 (1.1, 3.6) | 2.2 (1.1, 3.6) |

Values reported as median and 95 % credible interval

Estimated correlations for parameters *k*_avg_, *α*, *t*_0_, *k*_out_ and *IC*_50_ are presented in Table S3. For symmetry reasons, only the lower half of the resulting matrix is shown.

**Table S3** Estimated correlation parameters in **LL**^T^

|  | *k*_avg_ | *α* | *t*_0_ | *k*_out_ | *IC*_50_ |
| --- | --- | --- | --- | --- | --- |
| *k*_avg_ | 1.0 (1.0, 1.0) |  |  |  |  |
| *α* | -0.19 (-0.65, 0.38) | 1.0 (1.0, 1.0) |  |  |  |
| *t*_0_ | 0.086 (-0.59, 0.71) | -0.14 (-0.74, 0.59) | 1.0 (1.0, 1.0) |  |  |
| *k*_out_ | 0.12 (-0.52, 0.69) | -0.35 (-0.81, 0.25) | 0.13 (-0.58, 0.71) | 1.0 (1.0, 1.0) |  |
| *IC*_50_ | 0.097 (-0.54, 0.71) | -0.17 (-0.66, 0.40) | 0.095 (-0.60, 0.68) | 0.13 (-0.55, 0.74) | 1.0 (1.0, 1.0) |

Values reported as median and 95 % credible interval

## Model fits

Model fits for all horses are shown in Figs. S1 and S2.


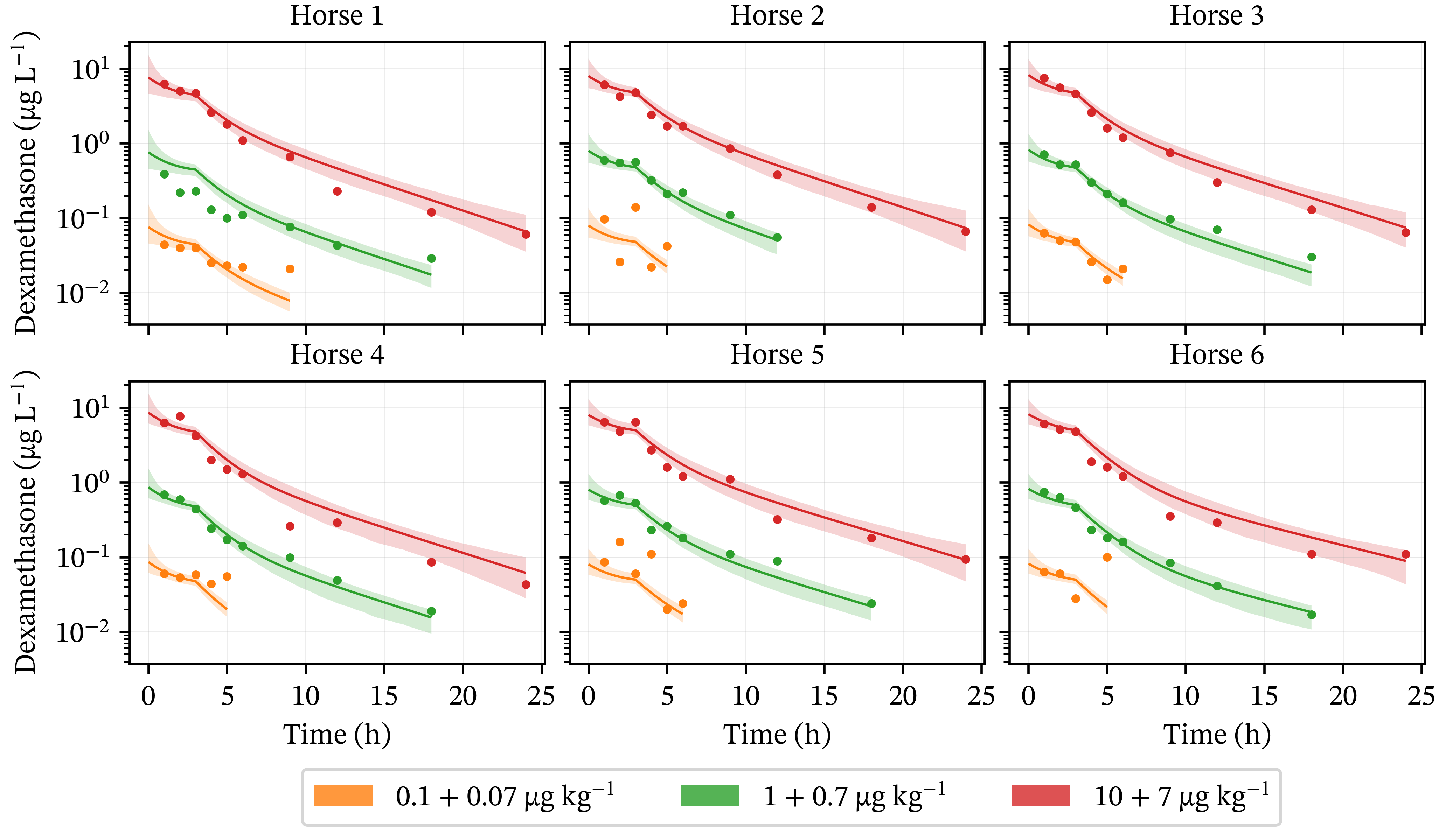


**Fig. S1** Model fits for dexamethasone concentration for all horses. Different dosing regimens are indicated by colour and respective dosing amounts are shown in the legend. Solid lines are time courses corresponding to the subject’s average parameters. Uncertainty in predicted time-courses is shown by shaded areas representing 95% of uncertainty.


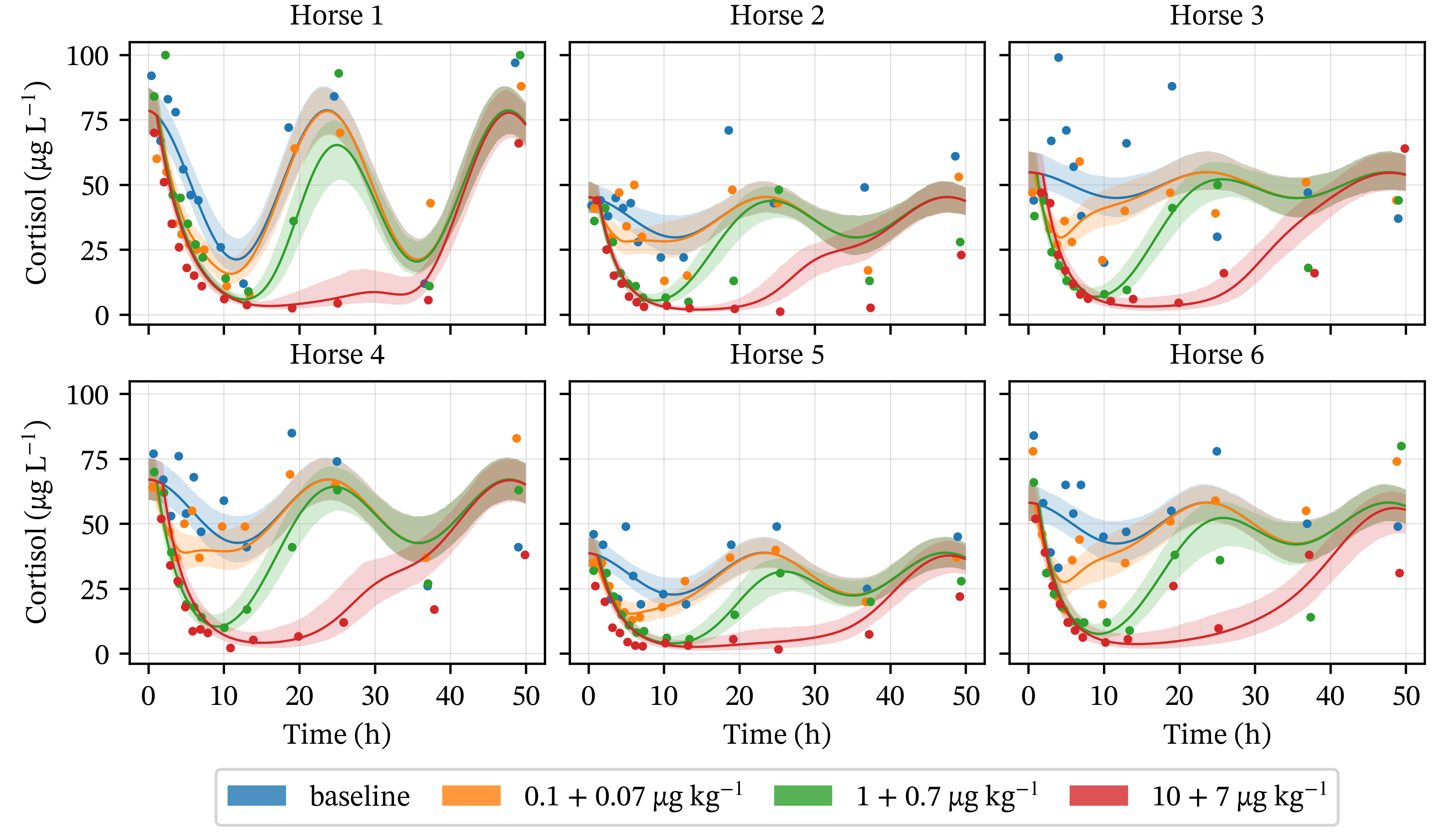


**Fig. S2** Model fits for cortisol concentrations for all horses. Different dosing regimens are indicated by colour and respective dosing amounts are shown in the legend. Solid lines are time courses corresponding to the subject’s average parameters. Uncertainty in predicted time-courses is shown by shaded areas representing 95% of uncertainty.

# Statistical modelling

## Choice of correlation parameters

For ease of modelling and to speed up parameter estimation, both the PK and the PD model were first formulated without the inclusion of modelled correlations between individual parameters. The marginal distributions for each parameter described in Section “Statistical parameter model” in the main article were the same as in the final model. Each sample from the posterior distribution gave a set of six individual parameters *θ_i_* for each parameter *θ* in the model. Pearson’s correlation coefficient was calculated between different model parameters *θ^k^* and *θ^l^* for each sample from the posterior distribution. This led to a distribution of correlation coefficients for each parameter combination. The median and 95 % credibility intervals of these distributions are shown in Tables 4, 5. Only six subjects were available in this study, so most correlations were deemed to be inestimable. Correlations were modelled when the credibility interval was sufficiently skewed to the positive or negative side. Respective rows were marked with a star.

Table S4: Quantiles of correlations in run without modelled correlation for PK parameters

| A | B | Median (95 % CI) | |  |
| --- | --- | --- | --- | --- |
| *Cl* | *Cl_d_* | 0.17 | (-0.69, 0.88) |  |
| *Cl* | *V_c_* | 0.30 | (-0.75, 0.91) |  |
| *Cl* | *V_t_* | 0.10 | (-0.79, 0.85) |  |
| *Cl_d_* | *V_c_* | -0.12 | (-0.84, 0.81) |  |
| *Cl_d_* | *V_t_* | 0.16 | (-0.76, 0.88) |  |
| *V_c_* | *V_t_* | -0.04 | (-0.81, 0.81) |  |

Correlations showed no clear shift towards positive or negative values for PK parameters in Table S4.

Table S5: Quantiles of correlations in run without modelled correlation for PD parameters

| A | B | Median (95 % CI) | |  |
| --- | --- | --- | --- | --- |
| *k_avg_* | *α* | -0.40 | (-0.76, 0.12) | * |
| *k_avg_* | *t*_0_ | 0.23 | (-0.75, 0.87) |  |
| *k_avg_* | *k_out_* | 0.42 | (-0.37, 0.83) | * |
| *k_avg_* | *I*_max_ | 0.04 | (-0.77, 0.82) |  |
| *k_avg_* | *IC*_50_ | 0.21 | (-0.37, 0.79) | * |
| *k_avg_* | *n* | -0.02 | (-0.82, 0.77) |  |
| *α* | *t*_0_ | -0.20 | (-0.86, 0.72) |  |
| *α* | *k_out_* | -0.64 | (-0.92, -0.14) | * |
| *α* | *I*_max_ | 0.15 | (-0.77, 0.79) |  |
| *α* | *IC*_50_ | -0.44 | (-0.84, 0.08) | * |
| *α* | *n* | 0.05 | (-0.69, 0.80) |  |
| *t*_0_ | *k_out_* | 0.21 | (-0.76, 0.87) |  |
| *t*_0_ | *I*_max_ | -0.03 | (-0.83, 0.81) |  |
| *t*_0_ | *IC*_50_ | 0.14 | (-0.72, 0.83) |  |
| *t*_0_ | *n* | -0.04 | (-0.85, 0.79) |  |
| *k_out_* | *I*_max_ | -0.19 | (-0.86, 0.75) |  |
| *k_out_* | *IC*_50_ | 0.43 | (-0.25, 0.91) | * |
| *k_out_* | *n* | -0.11 | (-0.81, 0.77) |  |
| *I*_max_ | *IC*_50_ | 0.19 | (-0.75, 0.80) |  |
| *I*_max_ | *n* | 0.03 | (-0.80, 0.84) |  |
| *IC*_50_ | *n* | 0.30 | (-0.67, 0.88) |  |

Correlations between *k*_avg_, *α*, *k*_out_ and *IC*_50_ found in Table S5 were included after this analysis. *t*_0_ was added so that all parameters in the oscillating turnover rate were modelled with correlations.

# Convergence diagnostics

## Monte Carlo error, effective sample size and Gelman-Rubin statistic

Summary of key diagnostics per parameter for the MCMC runs from Stan. Mean values, Monte Carlo standard errors, the standard deviation of the posterior distribution, effective sample size based on a MC sample of *N = 1000* and the Gelman-Rubin *R̂* statistic are shown in Tables S6 and S7. A detailed description of how these numbers were calculated can be found in Stan’s reference manual [1] p. 163–172. Note that it is possible that the effective sample size is larger than 1000 due to antithetical sampling (see Stan’s reference manual [1]). The diagnostics above are shown for all population hyper-parameters, individual parameters, and standard deviation(s) of the residual error variance model. As can be seen *R̂* values are very close to 1 for all parameters, indicating well-mixed MCMC chains. Monte Carlo standard errors are small and at least an order of magnitude smaller than the spread of present in the posterior distribution, as can be seen by comparing values of the MC standard error (se_mean) to the standard deviation of the posterior distribution (sd). Effective sample sizes are all larger than 200 and, in some cases, even larger than the size of the MCMC sample (*N = 1000*).

**Table S6** Parameter summaries for all parameters estimated in dexamethasone model

| Parameter | mean | se_mean | sd | n_eff | Rhat |
| --- | --- | --- | --- | --- | --- |
| mu_cl | 0.01 | 0.00 | 0.06 | 721.39 | 1.00 |
| mu_cld | -0.12 | 0.02 | 0.37 | 612.86 | 1.00 |
| mu_vc | 0.39 | 0.01 | 0.20 | 426.77 | 1.00 |
| mu_vt | -0.13 | 0.01 | 0.18 | 773.42 | 1.00 |
| tau_cl | 0.08 | 0.00 | 0.06 | 330.60 | 1.02 |
| tau_cld | 0.41 | 0.01 | 0.21 | 433.63 | 1.00 |
| tau_vc | 0.18 | 0.00 | 0.13 | 654.48 | 1.01 |
| tau_vt | 0.17 | 0.00 | 0.13 | 775.27 | 1.00 |
| eps | 0.48 | 0.00 | 0.03 | 942.34 | 1.00 |
| cl[1] | 0.53 | 0.00 | 0.04 | 570.38 | 1.01 |
| cl[2] | 0.49 | 0.00 | 0.03 | 1076.03 | 1.00 |
| cl[3] | 0.50 | 0.00 | 0.03 | 1003.71 | 1.00 |
| cl[4] | 0.51 | 0.00 | 0.04 | 874.02 | 1.00 |
| cl[5] | 0.47 | 0.00 | 0.04 | 521.02 | 1.01 |
| cl[6] | 0.49 | 0.00 | 0.03 | 919.82 | 1.00 |
| cld[1] | 0.23 | 0.00 | 0.12 | 848.40 | 1.00 |
| cld[2] | 0.21 | 0.00 | 0.12 | 633.08 | 1.00 |
| cld[3] | 0.20 | 0.00 | 0.10 | 837.76 | 1.00 |
| cld[4] | 0.17 | 0.00 | 0.09 | 737.70 | 1.00 |
| cld[5] | 0.18 | 0.00 | 0.09 | 753.71 | 1.00 |
| cld[6] | 0.13 | 0.00 | 0.06 | 503.63 | 1.00 |
| vc[1] | 1.32 | 0.02 | 0.37 | 383.69 | 1.01 |
| vc[2] | 1.26 | 0.01 | 0.28 | 636.97 | 1.00 |
| vc[3] | 1.22 | 0.01 | 0.26 | 763.58 | 1.00 |
| vc[4] | 1.17 | 0.01 | 0.24 | 667.42 | 1.00 |
| vc[5] | 1.25 | 0.01 | 0.25 | 557.49 | 1.00 |
| vc[6] | 1.23 | 0.01 | 0.23 | 758.58 | 1.00 |
| vt[1] | 0.82 | 0.01 | 0.19 | 775.93 | 1.00 |
| vt[2] | 0.75 | 0.01 | 0.19 | 1151.34 | 1.00 |
| vt[3] | 0.79 | 0.01 | 0.17 | 1018.11 | 1.00 |
| vt[4] | 0.73 | 0.00 | 0.16 | 1127.67 | 1.00 |
| vt[5] | 0.77 | 0.01 | 0.18 | 980.56 | 1.00 |
| vt[6] | 0.79 | 0.01 | 0.19 | 1191.84 | 1.00 |

**Table S7** Parameter summaries for all parameters estimated in cortisol model

| \| Parameter \| mean \| se_mean \| sd \| n_eff \| Rhat \| \| --- \| --- \| --- \| --- \| --- \| --- \| \| mu_kavg \| -0.28 \| 0.01 \| 0.18 \| 447.80 \| 1.00 \| \| mu_alpha \| 0.00 \| 0.02 \| 0.55 \| 512.65 \| 1.00 \| \| mu_t0 \| 0.22 \| 0.01 \| 0.13 \| 680.14 \| 1.00 \| \| mu_kout \| -0.51 \| 0.01 \| 0.13 \| 516.01 \| 1.00 \| \| mu_Imax \| 2.79 \| 0.03 \| 0.47 \| 274.07 \| 1.01 \| \| tau_Imax \| 0.43 \| 0.01 \| 0.22 \| 300.62 \| 1.01 \| \| mu_IC50 \| 0.78 \| 0.01 \| 0.24 \| 578.81 \| 1.00 \| \| mu_hill \| 1.64 \| 0.03 \| 0.48 \| 333.78 \| 1.01 \| \| tau_hill \| 0.43 \| 0.02 \| 0.30 \| 231.89 \| 1.01 \| \| tau[1] \| 0.37 \| 0.01 \| 0.13 \| 555.86 \| 1.00 \| \| tau[2] \| 1.15 \| 0.01 \| 0.24 \| 1285.43 \| 1.00 \| \| tau[3] \| 0.13 \| 0.00 \| 0.10 \| 830.63 \| 1.00 \| \| tau[4] \| 0.22 \| 0.00 \| 0.10 \| 532.98 \| 1.01 \| \| tau[5] \| 0.57 \| 0.01 \| 0.20 \| 665.33 \| 1.00 \| \| L[2,1] \| -0.17 \| 0.01 \| 0.28 \| 894.93 \| 1.00 \| \| L[2,2] \| 0.94 \| 0.00 \| 0.07 \| 653.00 \| 1.00 \| \| L[3,1] \| 0.09 \| 0.01 \| 0.36 \| 1068.27 \| 1.00 \| \| L[3,2] \| -0.10 \| 0.01 \| 0.34 \| 1216.13 \| 1.00 \| \| L[3,3] \| 0.85 \| 0.01 \| 0.13 \| 456.49 \| 1.01 \| \| L[4,1] \| 0.12 \| 0.01 \| 0.33 \| 954.61 \| 1.00 \| \| L[4,2] \| -0.32 \| 0.01 \| 0.28 \| 865.35 \| 1.00 \| \| L[4,3] \| 0.06 \| 0.01 \| 0.34 \| 796.25 \| 1.00 \| \| L[4,4] \| 0.75 \| 0.01 \| 0.15 \| 598.85 \| 1.01 \| \| L[5,1] \| 0.09 \| 0.01 \| 0.33 \| 1004.62 \| 1.00 \| \| L[5,2] \| -0.15 \| 0.01 \| 0.28 \| 811.73 \| 1.00 \| \| L[5,3] \| 0.06 \| 0.01 \| 0.36 \| 679.59 \| 1.00 \| \| L[5,4] \| 0.07 \| 0.01 \| 0.35 \| 658.69 \| 1.00 \| \| L[5,5] \| 0.70 \| 0.01 \| 0.17 \| 615.00 \| 1.01 \| \| hill[1] \| 2.50 \| 0.03 \| 0.60 \| 514.18 \| 1.01 \| \| hill[2] \| 2.69 \| 0.03 \| 0.67 \| 548.18 \| 1.00 \| \| hill[3] \| 2.56 \| 0.03 \| 0.62 \| 449.40 \| 1.00 \| \| hill[4] \| 2.43 \| 0.03 \| 0.62 \| 487.03 \| 1.00 \| \| hill[5] \| 2.31 \| 0.03 \| 0.65 \| 358.14 \| 1.00 \| \| hill[6] \| 2.21 \| 0.03 \| 0.62 \| 358.05 \| 1.01 \| | \| Parameter \| mean \| se_mean \| sd \| n_eff \| Rhat \| \| --- \| --- \| --- \| --- \| --- \| --- \| \| kavg[1] \| 13.27 \| 0.07 \| 1.61 \| 614.17 \| 1.00 \| \| kavg[2] \| 14.34 \| 0.07 \| 1.99 \| 782.15 \| 1.00 \| \| kavg[3] \| 19.29 \| 0.13 \| 3.11 \| 580.45 \| 1.00 \| \| kavg[4] \| 18.19 \| 0.09 \| 2.91 \| 950.17 \| 1.00 \| \| kavg[5] \| 10.70 \| 0.08 \| 1.88 \| 531.10 \| 1.00 \| \| kavg[6] \| 19.05 \| 0.17 \| 3.89 \| 514.42 \| 1.00 \| \| alpha[1] \| 0.80 \| 0.00 \| 0.08 \| 1146.64 \| 1.00 \| \| alpha[2] \| 0.26 \| 0.00 \| 0.09 \| 1091.74 \| 1.00 \| \| alpha[3] \| 0.12 \| 0.00 \| 0.06 \| 1614.47 \| 1.00 \| \| alpha[4] \| 0.29 \| 0.00 \| 0.08 \| 700.76 \| 1.00 \| \| alpha[5] \| 0.33 \| 0.00 \| 0.09 \| 1244.97 \| 1.00 \| \| alpha[6] \| 0.19 \| 0.00 \| 0.07 \| 1111.84 \| 1.00 \| \| t0[1] \| -3.33 \| 0.02 \| 0.53 \| 980.95 \| 1.00 \| \| t0[2] \| -2.77 \| 0.03 \| 0.94 \| 813.92 \| 1.01 \| \| t0[3] \| -2.62 \| 0.04 \| 1.18 \| 955.30 \| 1.00 \| \| t0[4] \| -2.75 \| 0.03 \| 0.88 \| 1011.92 \| 1.00 \| \| t0[5] \| -3.16 \| 0.03 \| 0.86 \| 940.66 \| 1.00 \| \| t0[6] \| -2.69 \| 0.04 \| 1.10 \| 830.10 \| 1.00 \| \| kout[1] \| 0.27 \| 0.00 \| 0.02 \| 475.16 \| 1.00 \| \| kout[2] \| 0.39 \| 0.00 \| 0.05 \| 601.35 \| 1.00 \| \| kout[3] \| 0.39 \| 0.00 \| 0.05 \| 569.48 \| 1.00 \| \| kout[4] \| 0.33 \| 0.00 \| 0.05 \| 728.81 \| 1.00 \| \| kout[5] \| 0.35 \| 0.00 \| 0.05 \| 469.72 \| 1.00 \| \| kout[6] \| 0.38 \| 0.00 \| 0.07 \| 492.32 \| 1.00 \| \| Imax[1] \| 0.95 \| 0.00 \| 0.02 \| 472.00 \| 1.00 \| \| Imax[2] \| 0.95 \| 0.00 \| 0.02 \| 353.88 \| 1.00 \| \| Imax[3] \| 0.94 \| 0.00 \| 0.03 \| 377.95 \| 1.01 \| \| Imax[4] \| 0.94 \| 0.00 \| 0.04 \| 398.23 \| 1.00 \| \| Imax[5] \| 0.92 \| 0.00 \| 0.05 \| 372.88 \| 1.00 \| \| Imax[6] \| 0.93 \| 0.00 \| 0.04 \| 359.44 \| 1.00 \| \| IC50[1] \| 0.03 \| 0.00 \| 0.01 \| 857.30 \| 1.00 \| \| IC50[2] \| 0.06 \| 0.00 \| 0.01 \| 1026.05 \| 1.00 \| \| IC50[3] \| 0.04 \| 0.00 \| 0.01 \| 785.36 \| 1.00 \| \| IC50[4] \| 0.05 \| 0.00 \| 0.01 \| 1137.72 \| 1.00 \| \| IC50[5] \| 0.03 \| 0.00 \| 0.01 \| 639.88 \| 1.00 \| \| IC50[6] \| 0.04 \| 0.00 \| 0.01 \| 851.49 \| 1.00 \| \| sigma_add \| 1.00 \| 0.02 \| 0.70 \| 1038.24 \| 1.00 \| \| sigma_prop \| 0.31 \| 0.00 \| 0.02 \| 1430.06 \| 1.00 \| |
| --- | --- | --- | --- | --- | --- | --- | --- | --- | --- | --- | --- | --- | --- | --- | --- | --- | --- | --- | --- | --- | --- | --- | --- | --- | --- | --- | --- | --- | --- | --- | --- | --- | --- | --- | --- | --- | --- | --- | --- | --- | --- | --- | --- | --- | --- | --- | --- | --- | --- | --- | --- | --- | --- | --- | --- | --- | --- | --- | --- | --- | --- | --- | --- | --- | --- | --- | --- | --- | --- | --- | --- | --- | --- | --- | --- | --- | --- | --- | --- | --- | --- | --- | --- | --- | --- | --- | --- | --- | --- | --- | --- | --- | --- | --- | --- | --- | --- | --- | --- | --- | --- | --- | --- | --- | --- | --- | --- | --- | --- | --- | --- | --- | --- | --- | --- | --- | --- | --- | --- | --- | --- | --- | --- | --- | --- | --- | --- | --- | --- | --- | --- | --- | --- | --- | --- | --- | --- | --- | --- | --- | --- | --- | --- | --- | --- | --- | --- | --- | --- | --- | --- | --- | --- | --- | --- | --- | --- | --- | --- | --- | --- | --- | --- | --- | --- | --- | --- | --- | --- | --- | --- | --- | --- | --- | --- | --- | --- | --- | --- | --- | --- | --- | --- | --- | --- | --- | --- | --- | --- | --- | --- | --- | --- | --- | --- | --- | --- | --- | --- | --- | --- | --- | --- | --- | --- | --- | --- | --- | --- | --- | --- | --- | --- | --- | --- | --- | --- | --- | --- | --- | --- | --- | --- | --- | --- | --- | --- | --- | --- | --- | --- | --- | --- | --- | --- | --- | --- | --- | --- | --- | --- | --- | --- | --- | --- | --- | --- | --- | --- | --- | --- | --- | --- | --- | --- | --- | --- | --- | --- | --- | --- | --- | --- | --- | --- | --- | --- | --- | --- | --- | --- | --- | --- | --- | --- | --- | --- | --- | --- | --- | --- | --- | --- | --- | --- | --- | --- | --- | --- | --- | --- | --- | --- | --- | --- | --- | --- | --- | --- | --- | --- | --- | --- | --- | --- | --- | --- | --- | --- | --- | --- | --- | --- | --- | --- | --- | --- | --- | --- | --- | --- | --- | --- | --- | --- | --- | --- | --- | --- | --- | --- | --- | --- | --- | --- | --- | --- | --- | --- | --- | --- | --- | --- | --- | --- | --- | --- | --- | --- | --- | --- | --- | --- | --- | --- | --- | --- | --- | --- | --- | --- | --- | --- | --- | --- | --- | --- | --- | --- | --- | --- | --- | --- | --- | --- | --- | --- | --- | --- | --- | --- | --- | --- | --- | --- | --- | --- | --- | --- | --- | --- | --- | --- | --- | --- | --- | --- | --- | --- | --- | --- | --- | --- | --- | --- | --- | --- | --- | --- | --- | --- | --- | --- | --- | --- | --- | --- | --- | --- | --- | --- | --- | --- | --- | --- | --- | --- | --- | --- | --- | --- | --- | --- | --- | --- | --- | --- | --- | --- | --- | --- | --- | --- | --- | --- |

## Posterior predictive plots

Posterior predictive plots [2] simulate new data based on the full model. Therefore, parameter uncertainty and residual error variance are used to simulate new data and the results for the dexamethasone exposure model and the cortisol turnover model are shown below. Shaded uncertainty intervals show 95 % intervals and should therefore include about 95 % of all data points for the model to be reasonable. As can be seen in Figs. S3 and S4 most data points are inside the simulated uncertainty bounds and only few are outside, indicating a reasonable description of the data through the model.


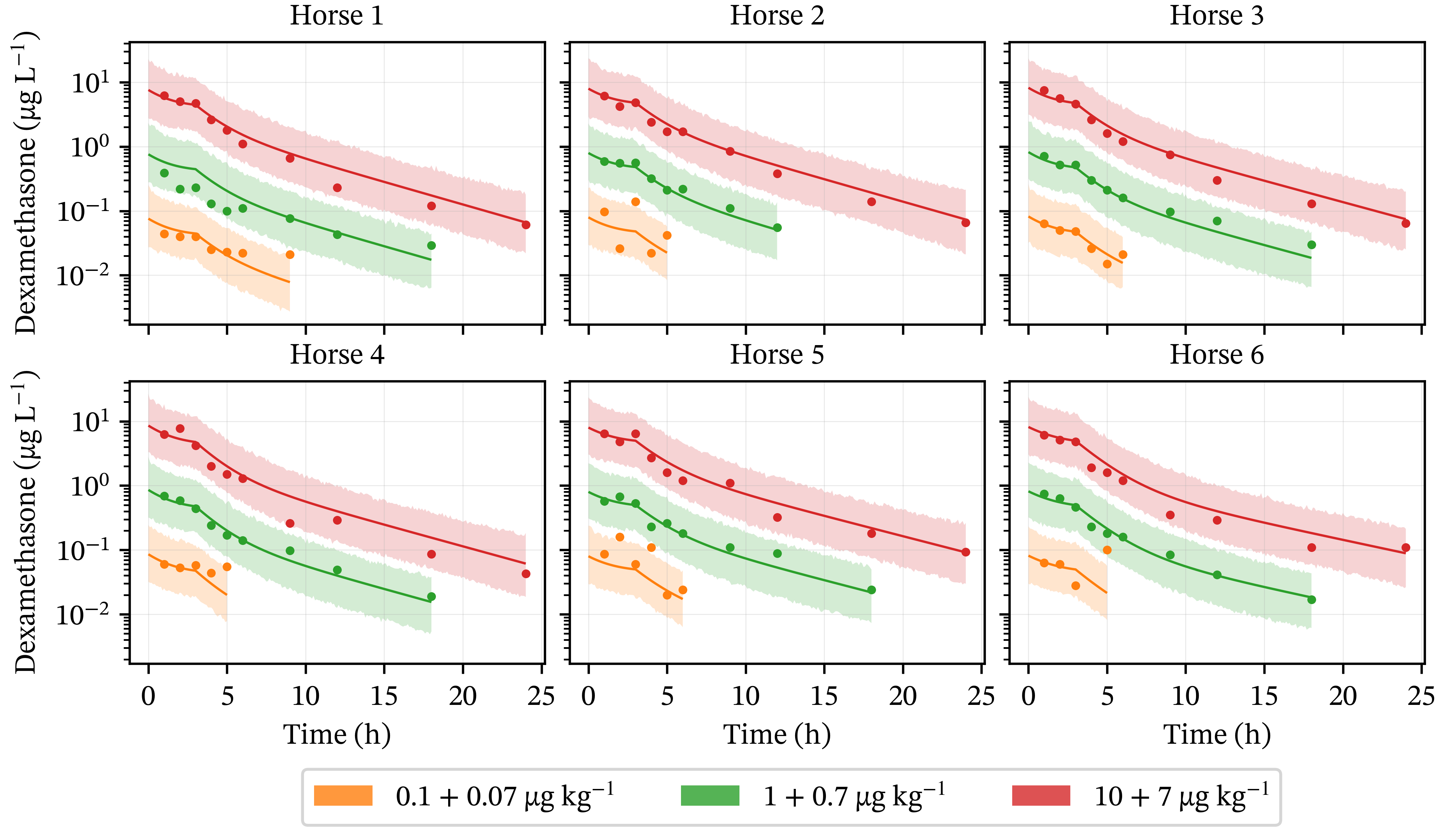


**Fig. S3** Posterior predictive plot for dexamethasone concentrations. Different dosing regimens are indicated by colour and respective dosing amounts are shown in the legend. Solid lines are time courses corresponding to the subject’s average parameters. Uncertainty in predicted time-courses is shown by shaded areas representing 95% of uncertainty.


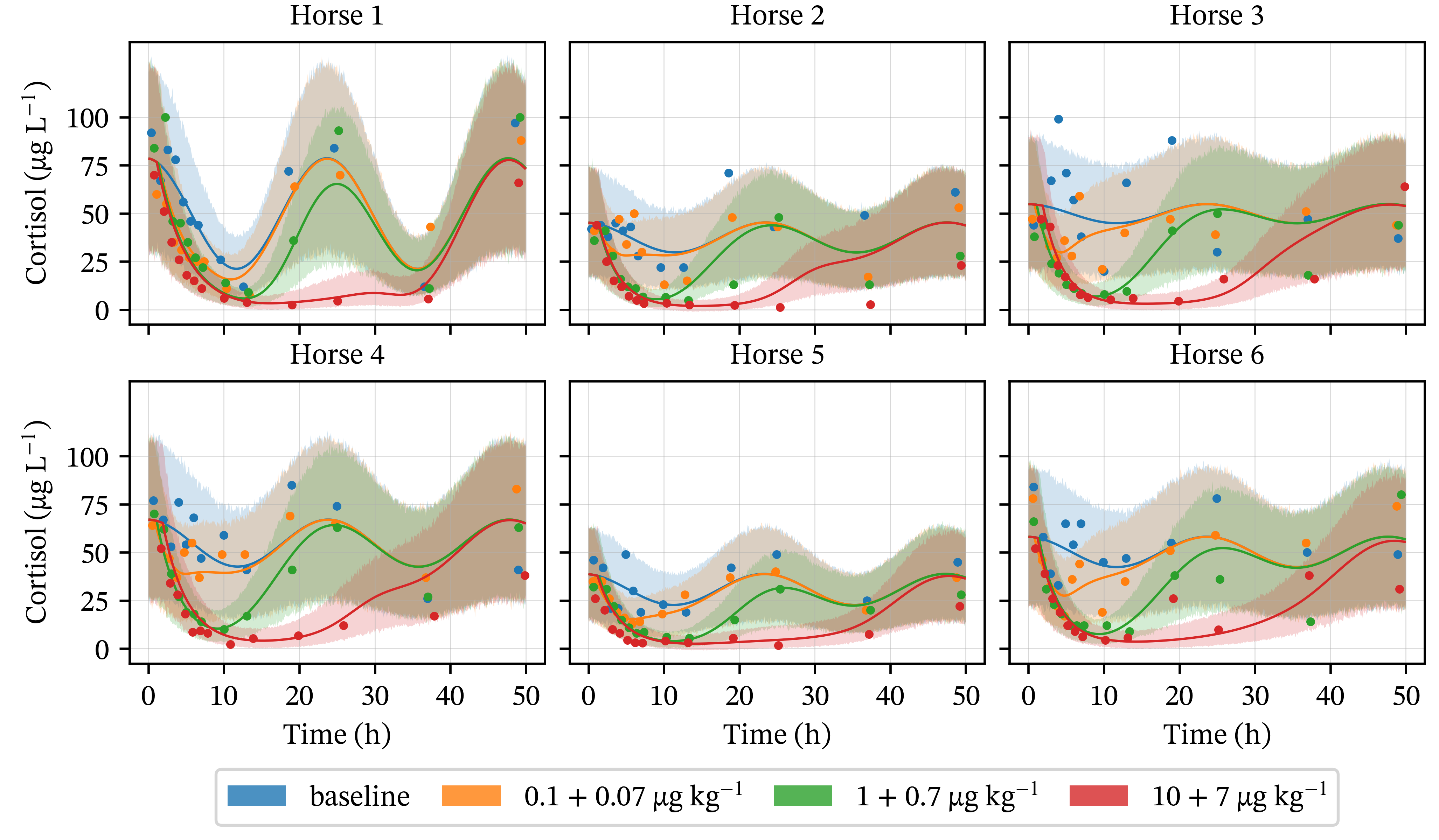


**Fig. S4** Posterior predictive plot for cortisol concentrations. Different dosing regimens are indicated by colour and respective dosing amounts are shown in the legend. Solid lines are time courses corresponding to the subject’s average parameters. Uncertainty in predicted time-courses is shown by shaded areas representing 95% of uncertainty.


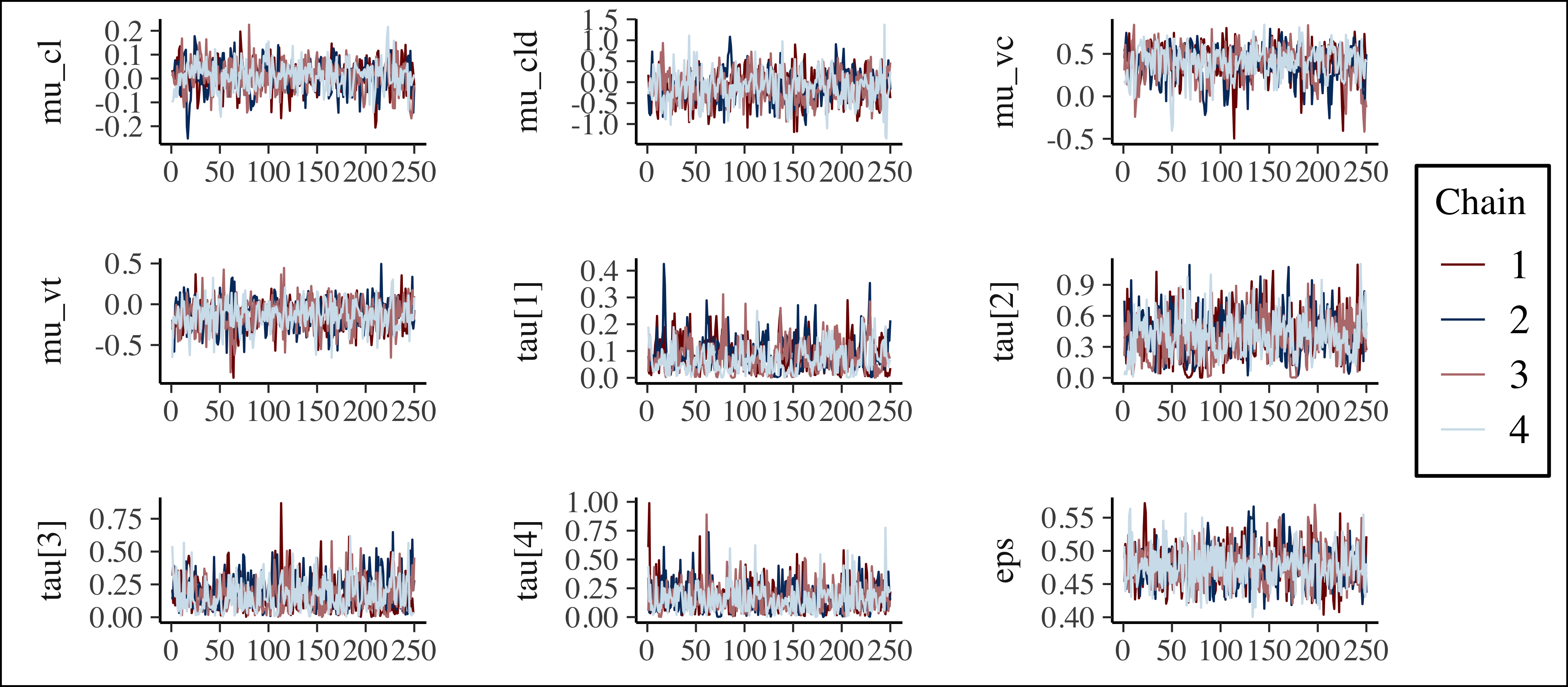


**Fig. S5** Trace plots for population parameters of the dexamethasone model. Different colours indicated different chains as noted in the legend

## Trace plots

Trace plots for the population hyperparameters and standard deviation(s) of the residual error variance models are shown below. These show no apparent trend or regular pattern and indicate therefore good mixing and convergence of the Markov chains. Note that each chain is started at different random initial values. All chains converge to the same posterior distribution, indicating that the posterior distribution is unimodal, which is important for the proper functioning of MCMC methods. Trace plots for the population parameters of the dexamethasone model can be seen in Fig. S5 and for the cortisol model in Fig. S6.


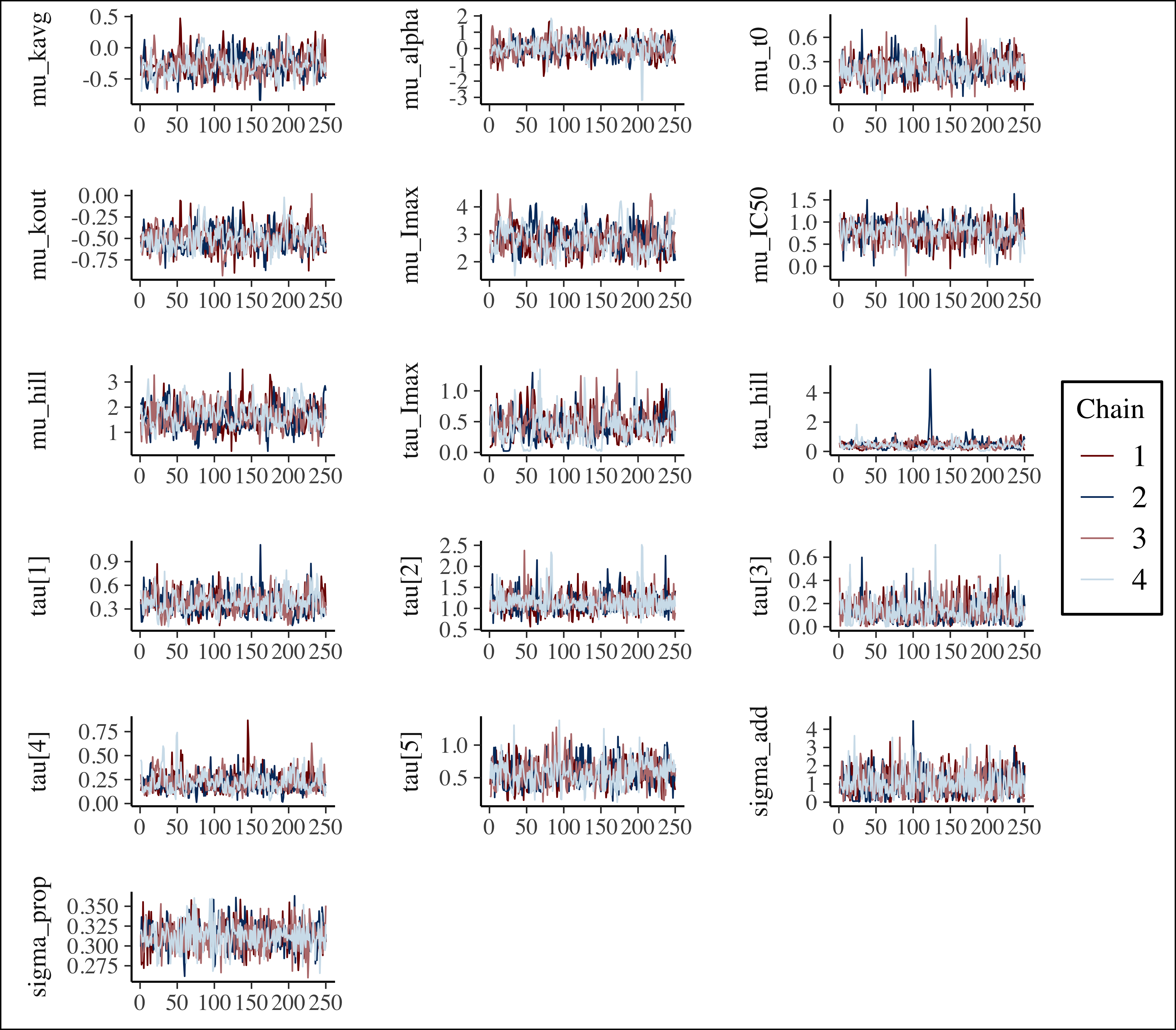


**Fig. S6** Trace plots for population parameters of the cortisol model. Different colours indicated different chains as noted in the legend

## Hamiltonian Monte Carlo energy

The energy Bayesian fraction of missing information (E-BFMI) was checked to ensure proper exploration of the parameter space. The theoretical background of this criterion is elaborate [3], but in practice it can easily be checked by comparison of two histograms as shown in Figs. S7 and S8. The better the two histograms overlap, the more certainty there is that the Markov chains did not miss a substantial part of the parameter space. As can be seen in the figures below, the histograms overlap for the most part and there are no apparent patterns or systematic mismatches.


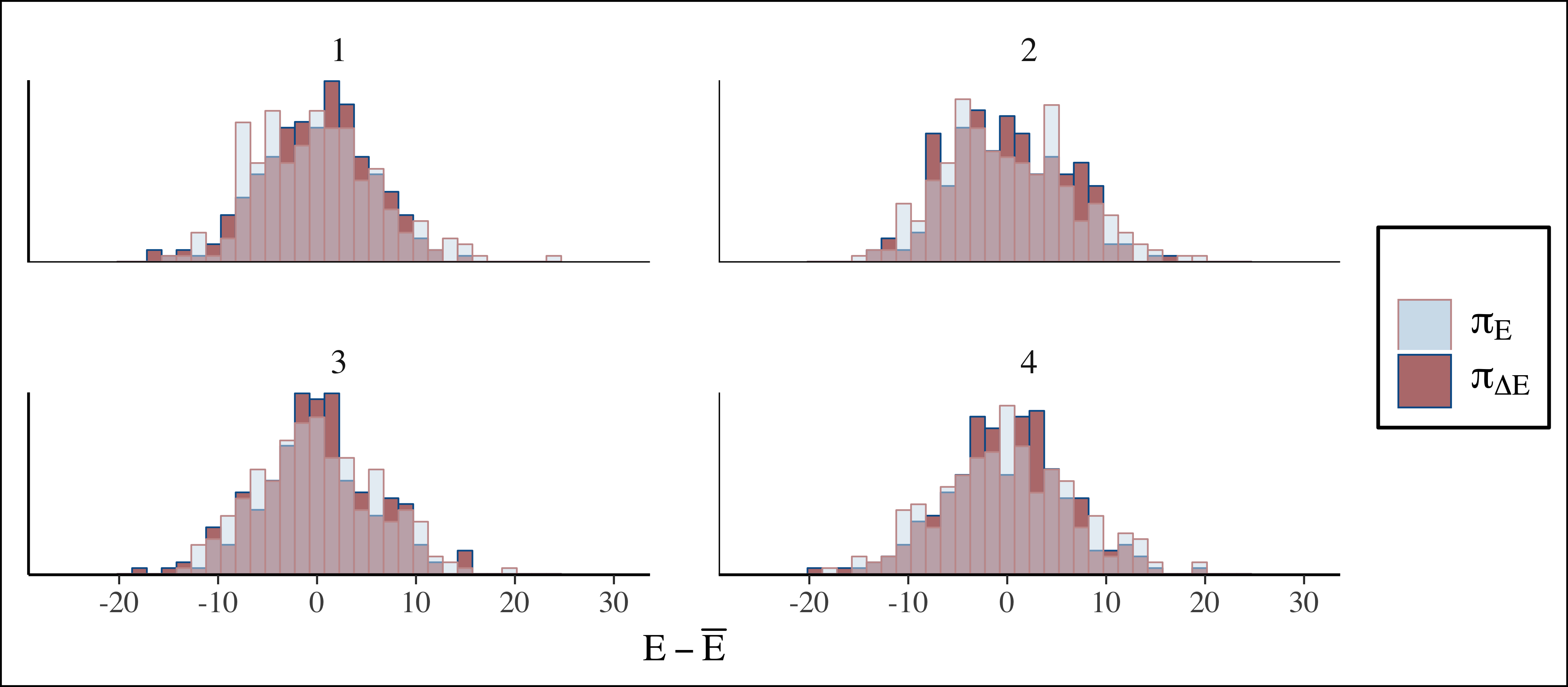


**Fig. S7** Energy histogram for the four chains sampling from the dexamethasone model


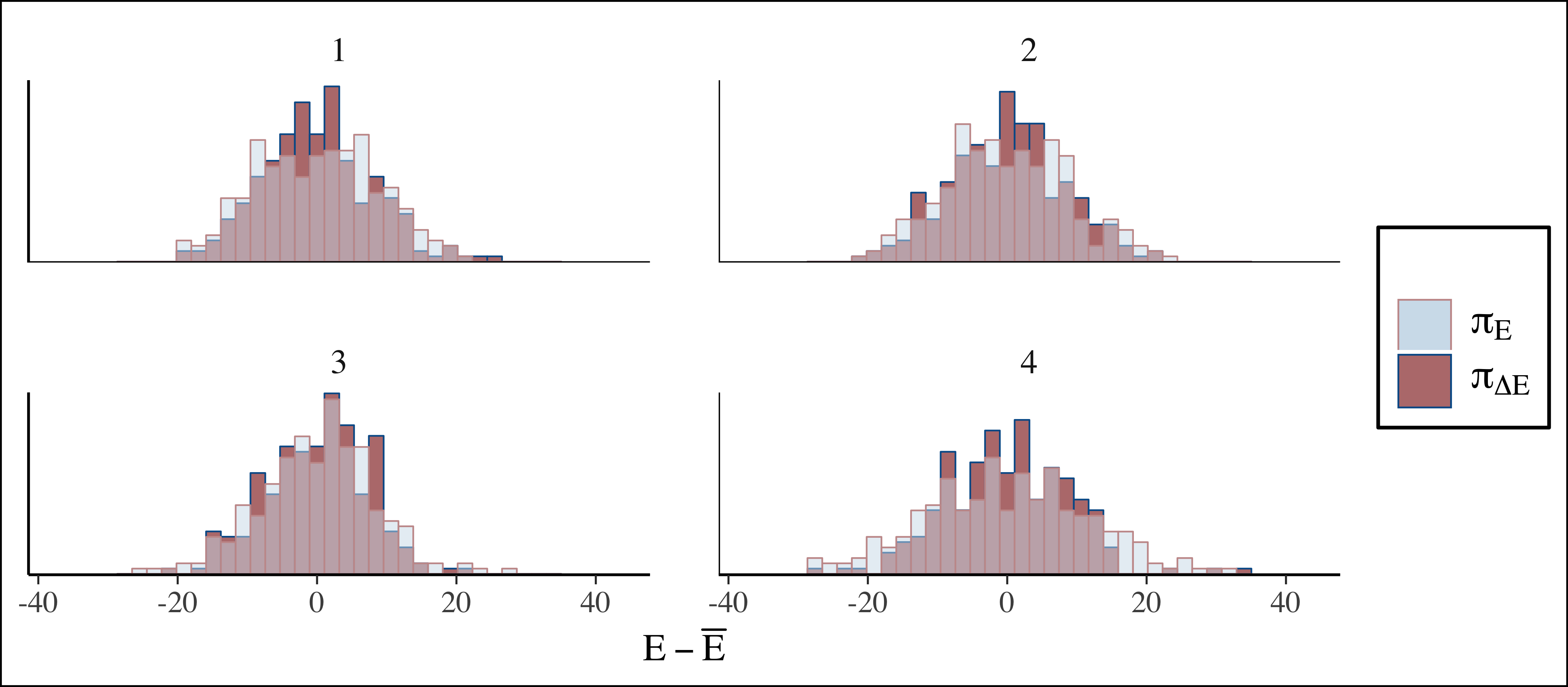


**Fig. S8** Energy histogram for the four chains sampling from the cortisol model

# References

1. Stan Development Team (2018) Stan: Reference manual v2.18.0. <https://github.com/stan-dev/stan/releases/download/v2.18.0/reference-manual-2.18.0.pdf>. Accessed 28 Nov 2018

2. Gelman A, Carlin JB, Stern HS, Dunson DB, Vehtari A, Rubin DB (2013) Bayesian Data Analysis, 3rd edn. CRC Press, Boca Raton, Florida

3. Betancourt M (2018) A Conceptual Introduction to Hamiltonian Monte Carlo. arXiv pre-print. <https://arxiv.org/pdf/1701.02434.pdf>. Accessed 29 Aug 2018
